# High-throughput automated microfluidic sample preparation for accurate microbial genomics

**DOI:** 10.1038/ncomms13919

**Published:** 2017-01-27

**Authors:** Soohong Kim, Joachim De Jonghe, Anthony B. Kulesa, David Feldman, Tommi Vatanen, Roby P. Bhattacharyya, Brittany Berdy, James Gomez, Jill Nolan, Slava Epstein, Paul C. Blainey

**Affiliations:** 1 The Broad Institute of MIT and Harvard, Cambridge, Massachusetts 02142, USA; 2 Department of Biological Engineering, Massachusetts Institute of Technology, Cambridge, Massachusetts 02139, USA; 3 Department of Biochemistry, University of Cambridge, Cambridge CB2 1GA, UK; 4 Department of Physics, Massachusetts Institute of Technology, Cambridge, Massachusetts 02139, USA; 5 Department of Computer Science, Aalto University School of Science, Espoo 02150, Finland; 6 Division of Infectious Diseases, Massachusetts General Hospital, Boston, Massachusetts 02114, USA; 7 Department of Biology, Northeastern University, Boston, Massachusetts 02115, USA

## Abstract

Low-cost shotgun DNA sequencing is transforming the microbial sciences. Sequencing instruments are so effective that sample preparation is now the key limiting factor. Here, we introduce a microfluidic sample preparation platform that integrates the key steps in cells to sequence library sample preparation for up to 96 samples and reduces DNA input requirements 100-fold while maintaining or improving data quality. The general-purpose microarchitecture we demonstrate supports workflows with arbitrary numbers of reaction and clean-up or capture steps. By reducing the sample quantity requirements, we enabled low-input (∼10,000 cells) whole-genome shotgun (WGS) sequencing of *Mycobacterium tuberculosis* and soil micro-colonies with superior results. We also leveraged the enhanced throughput to sequence ∼400 clinical *Pseudomonas aeruginosa* libraries and demonstrate excellent single-nucleotide polymorphism detection performance that explained phenotypically observed antibiotic resistance. Fully-integrated lab-on-chip sample preparation overcomes technical barriers to enable broader deployment of genomics across many basic research and translational applications.

## Abstract (web summary)

Shotgun DNA sequencing experiments for microbial genomic analysis are often impractical due to minimum sample input requirements. Here the authors develop a microfluidic sample preparation platform that reduces sample input requirements 100-fold and enables high throughput sequencing from low numbers of cells.

Low-cost DNA sequence data generation is enabling the widespread application of genomic methods across the microbial sciences. Genome sequencing can comprehensively survey commensal microbiota^1^, enable the diagnosis of drug resistant infections^2^,^3^,^4^,^5^,^6^ and reveal networks through which infections are transmitted^7^. In particular, pathogen surveillance by whole-genome shotgun (WGS) analysis provides information for molecular epidemiology of critical value to public health^7^,^8^ that cannot be obtained by culture or PCR. To this point, a recent Executive Order^9^ called for nationwide tracking of antibiotic resistance in microbial pathogens by genome sequencing in the US. In addition, natural products produced by microbes continue to serve as a rich source of therapeutic compounds spanning antibiotics to cancer^10^. Such compounds can be discovered by performing large-scale sequencing of environmental samples^11^.

Despite impressive progress in technology for sequence data production, the methods used to prepare sequencing samples lag behind (Supplementary Fig. 1). To sequence bacterial genomes, cells must be lysed and their DNA purified, fragmented, tagged with adaptors and size-selected before loading on a sequencing instrument. The complex experimental logistics and labour currently required to complete these steps limit sample throughput. The introduction of liquid handling robotics and electrowetting-based ‘digital' microfluidics have helped to increase throughput, but these workflows require high DNA input, do not integrate all the key workflow steps (variously omitting cell lysis, DNA fragmentation and size selection), and substantially offset reductions in reagent and labour costs with expensive proprietary equipment and consumables (Supplementary Fig. 2)^12^.

The performance of available sample preparation methods on low-quantity samples is limiting in many microbial applications, as microbes can be difficult to isolate and grow to the quantities required for sequencing. Data from samples that are expanded by extensive culture or biochemical amplification can be significantly biased and are more likely to be contaminated^13^,^14^,^15^. Available library construction techniques typically require inputs of >1 million cell equivalents and are susceptible to contamination (particularly at low-input levels)^16^,^17^,^18^.

Whole-genome sequencing holds promise as a low-cost, rapid, essentially universal diagnostic for infectious disease^19^,^20^,^21^. However, input quantity requirements present a serious barrier for rapid WGS-based diagnostics for slow-growing microbes like *Mycobacterium tuberculosis*. Applications in environmental microbiology and natural product discovery are even further restricted by high-input requirements since only about 1% of environmental microbial isolates are easily cultured to produce a large quantity of pure genomic material^22^. Innovations in environmental sampling and culturing such as the iChip^22^ enhance the chance of producing novel isolates, but also produce micro-colonies that are recalcitrant to scale-up culture and provide too little biomass for available direct WGS approaches.

Here we introduce a new poly dimethylsulfoxide (PDMS) microfluidic circuit architecture that integrates all the major steps in sample preparation for the first time (Supplementary Fig. 2; Supplementary Data 1) and makes major advances in input requirement and throughput while maintaining data quality. We demonstrated the new microarchitecture for sample preparation from clinical pathogen and environmental isolates, showing excellent data quality at extraordinarily low-input quantities. We expect this sample preparation platform will find wide application in a variety of high sample throughput genomics applications^2^,^3^,^4^,^5^,^23^,^24^,^25^,^26^.

## Results

### Microdevice design and operation

**Figure 1:**
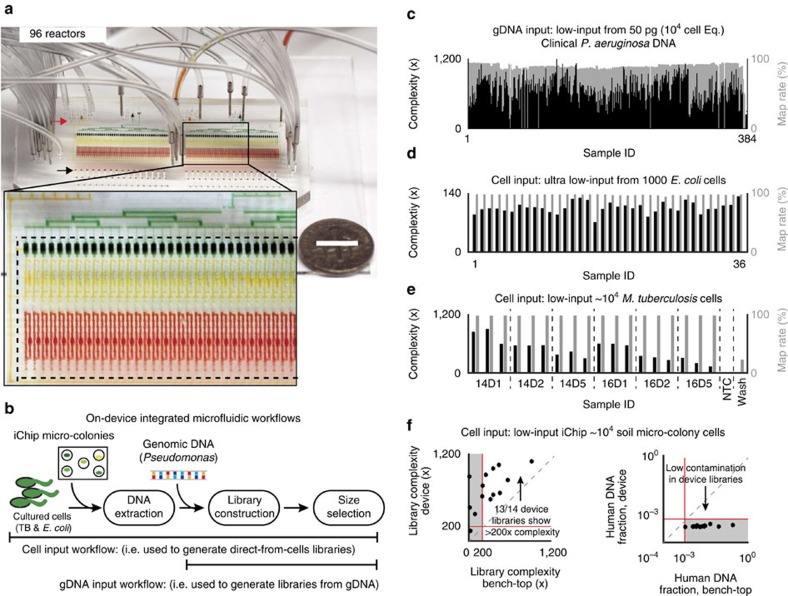
Genomic sample preparation device operation and performance. (**a**) A photograph of the 96 × 36 nl microfluidic sample preparation device filled with food colouring to highlight features. Dime indicates scale (white bar indicates 1 cm). Inset: the reactor (red), filter (yellow) and reservoir (green) units. Black and red arrows designate reagent input ports and sample input/output ports, respectively. (**b**) The microfluidic sample preparation workflows for biomass input (extreme left) and gDNA input (top). (**c**–**e**) Estimated sequence library complexity (units of fold-coverage) and mapping rate for (**c**) the clinical *P. aeruginosa* isolate gDNA samples mapped to *PA-14* (of the 384 samples, eight individual replicates were lost completely during the barcoding step, leaving two replicates for each of these isolates. We know that these PCR drop-outs occurred due to faulty primers because these particular eight primer sets were subsequently observed to fail consistently across multiple samples). (**d**) Low-input *E. coli* biomass samples mapped to *BL21-DE3* and (**e**) low-input *M. tuberculosis* biomass samples mapped to *OFXR-14*. (**f**) Comparison of microfluidic and optimized bench-top sample preparation from low-input from soil micro-colony biomass (left, library complexity; right, human contamination). The library complexities were calculated using Picard tools (http://broadinstitute.github.io/picard/) and the human DNA read fraction was determined using deconseq (http://deconseq.sourceforge.net/).

Microfluidics is a natural solution to increase throughput and reduce input requirements thanks to its scalable automation and capability to precisely manipulate small volumes^27^,^28^. Even small bench-top sequencing instruments like the MiSeq can sequence many microbial genomes per run, necessitating larger sample preparation batch sizes. Thus, we optimized a high-density two-layer microfluidic^27^ sample-processing microarchitecture that enables a batch size up to 96 samples per device run (Fig. 1 and Supplementary Fig. 3). Integrating all the key WGS sample preparation steps in our automated microfluidic device resulted in increased throughput, reduced reagent consumption, reduced input requirements, reduced contamination and improved reproducibility.

To prepare samples for genome sequencing, DNA must first be extracted from cells and then processed into properly sized fragments with attached sequencing adaptors. We extract genomic DNA in the device by lysing cells with a combination of heat, detergents, and hydrolytic enzymes. To fragment and attach adaptors to the gDNA, we apply the ‘tagmentation' chemistry^23^,^24^,^25^,^26^,^29^, which uses transposase enzymes to insert adaptor oligonucleotide sequences directly into high molecular weight DNA. For DNA purification, which is required at multiple points in the sample preparation process, we implement the solid phase reversible immobilization (SPRI)^30^ method in the microfluidic system (Supplementary Note 1).

As we increased the reactor density to fit 96 channels in a 70mm × 35mm device, we adapted our design to prevent cross contamination among samples and reagents, while still allowing for a single shared waste stream and individual collection of products.

Our microfluidic circuit performs the DNA extraction and library construction steps in a rotary reactor^31^ of 36 nanoliters (nl) that metres and mixes reagents and operates in concert with filter valves to strain cells or beads from solution (Fig. 1a and Supplementary Fig. 4). Standard micro-valves partition the rotary reactor for dead-end-fill loading^27^ of precise quantities of each reagent. These same valves are used coordinately as peristaltic pumps to mix reaction components within the rotary reactors (Supplementary Fig. 3a). Filter valves are improved sieve valves^27^ that can be actuated to rapidly strain particles from solution (Supplementary Fig. 5). To concentrate cells or purify, concentrate, and size-select nucleic acid products, we capture beads using filter valves at the output of each reactor (Supplementary Fig. 3a). This purification approach requires no chemical modification of the device^32^ and simplifies the reaction circuit since the filter valves can be directed to collect or release beads by flow in either direction, eliminating the need for additional bypass channels that reduce reactor density^33^. The micro-automated SPRI purifications used in DNA extraction, reaction clean-up and size selection operations yield >80% recovery of picogram-level starting material (Supplementary Fig. 3b).

To establish multiple manifolds for solution loading/unloading in the two-layer high-density format, we strategically placed waste port ‘vias' within each filter unit that connect to a ‘sewer line' in the bottom layer of the device^33^ (Supplementary Fig. 3a). This arrangement enables bead, sample and reagent wastes to escape the high-density reaction circuits in the device upper layer without cross contamination (Supplementary Fig. 6; Supplementary Note 2) and without the complexity of extra device layers or extra space needed for internal access ports^34^. We initially load reagents into each reactor through one manifold (red arrow, Fig. 1a), while cells or gDNA samples are loaded via individual input/output ports (black arrow, Fig. 1a).

Finally, the re-use of each rotary reactor for multiple process steps eliminates the need for a series of reactors matched to each workflow step. Re-use is enabled by the capability for reaction/pull-down (for example, filtration or purification) steps. The combination of reactor re-use for an arbitrary number of process steps together with pull-down/purification capability makes the system largely protocol-independent since most next-generation sequencing (NGS) and cell-handling processes can be accomplished with some number of these elementary steps in combination.

### Low-input DNA extraction and library construction

The extraction and library construction workflow uses each reactor for five different reaction steps (lysis, 2 × DNA precipitation, tagmentation and reaction stop) and the filter unit for three different capture steps (cell capture and 2 × SPRI bead capture). Low sample input capability requires high efficiency lysis, purification and tagmentation, as well as contamination resistance to prevent small samples from being overwhelmed by extraneous DNA. We validated our microfluidic platform by processing four different low-input samples; samples of ∼1,000 *E. coli* cells, ∼10,000 *M. tuberculosi*s cells, ∼10,000 cell soil micro-colony samples, and GC-rich genomic DNA samples from clinical *P. aeruginosa* isolates (Supplementary Data 2).

The microfluidic devices consistently converted 5–15% of input gDNA into library molecules as estimated by qPCR measurement of output tagmentation products (Supplementary Fig. 3c). This value is significantly higher than previously reported low-input library construction efficiencies of 0.5–2% (ref. 35). Efficiency estimates derived from the duplicate sequence read rate agreed with our qPCR analysis^36^ (Supplementary Note 3). Libraries constructed on-chip from 50–100 picograms gDNA input showed reproducible mapping rates, fragment sizes and insert sizes, and k-mer frequencies consistent with standard high-input sample preparation (Fig. 1c, Supplementary Figs 3d and 7)^37^.

On-device cell lysis and DNA extraction steps were also found to be highly efficient. We measured 10% end-to-end conversion efficiency and found good library quality when using an ultra-low input of just 1,000 *E. coli* cells, representing sample and reagent inputs 200 times lower than standard ‘low input' protocols that specify 1 ng input (Fig. 1d; Supplementary Note 3). High efficiency was also achieved for organisms like *M. tuberculosis* (10,000 cell input) and environmental micro-colonies that are known to be more challenging to lyse (Fig. 1e,f; Supplementary Note 4).

To test how contamination and sequence library quality compare at low input to conventional sample preparation, we carried out a matched comparison of environmental micro-colony sequence libraries prepared by the microfluidic method with those prepared on the bench-top by an optimized low-input procedure using the same reagents in the same laboratory environment by the same operator (Supplementary Note 5).

For this comparison, we used soil samples from a private Boston garden grown using the iChip^22^ system, which cultures environmental microbes *in situ* to produce micro-colonies (Supplementary Fig. 8). We estimated the iChip micro-colonies to contain on the order of 100,000 cells each in total, and the input for sample preparation to be on the order of 10,000 cells (Supplementary Note 4). Each sample was split in half. One part was directly loaded and processed in our device (Fig. 1b and Supplementary Fig. 9), while the remaining portion was processed using an equivalent sample-processing technique at the bench-top. Comparing data from the two sample preparation methods produced from the same micro-colonies in the same HiSeq 2500 sequencing run, the on-device sample preparation yielded an average of twice the library complexity, half the coefficient of variation in complexity, no drop-outs and far lower human DNA contamination (Fig. 1f).

### High-throughput library construction from clinical isolates

**Figure 2:**
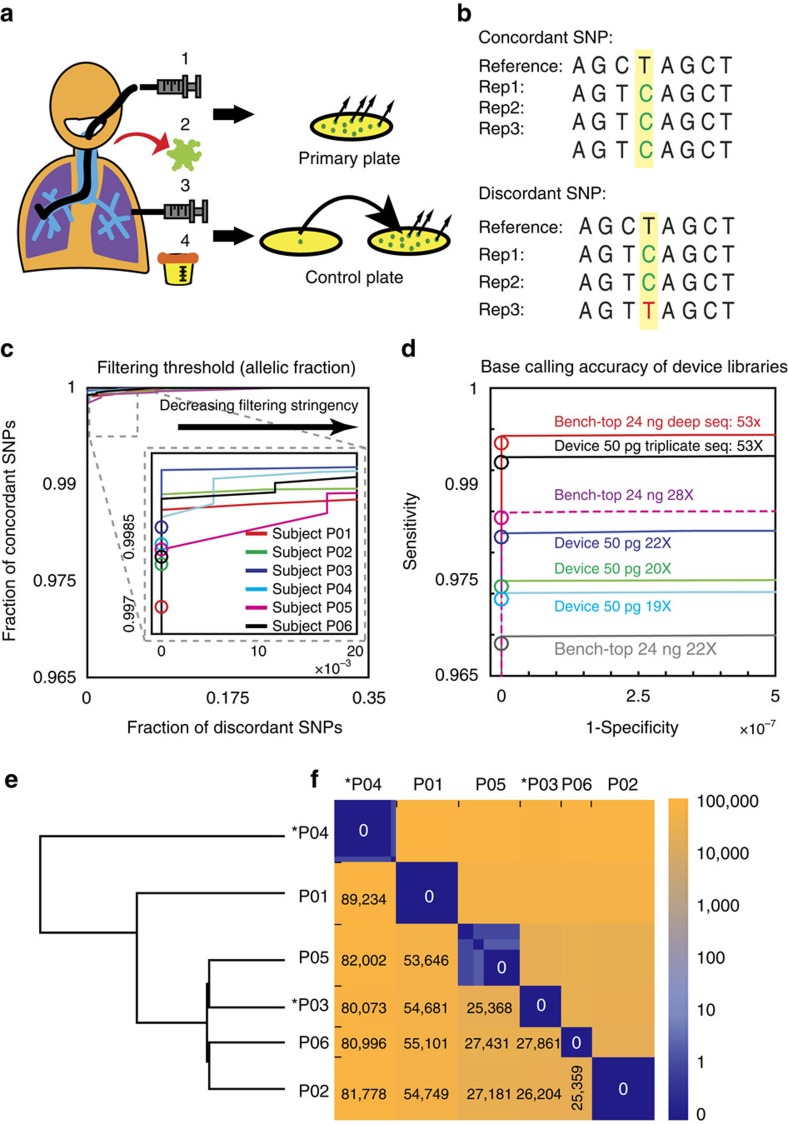
*Pseudomonas aeruginosa* clinical isolate sequencing and error correction. (**a**) Four types of samples were collected from six different subjects; (1) bronchial alveolar lavage, (2) sputum, (3) thoracostomy and (4) urine. Samples were streaked on primary plates and two control plates, which are expanded from single colonies. (**b**) Concordant SNP call sets are defined as call sets that agree among all technical replicate libraries. (**c**) The fraction of concordant and discordant SNPs among technical replicates (*n*=3) of one isolate from each subject (P01-4, P02-26, P03-58, P04-72, P05-105 and P06-115, where the notation P01-4 means patient subject 1, sample 4) is plotted with decreasing AF stringency (minimum read depth fixed at 6; minimum mapping quality fixed at 45; excluding indels; see ‘Methods' section). Inset: thresholding at an AF value of 0.82 (circles) balances the maximization of concordant SNPs and minimization of discordant SNPs and was used for variant calling across all our samples. (**d**) Receiver operator characteristic (ROC) plot compares the accuracy and sensitivity in SNP calling among low-input libraries of sample P01-4 made in the device, on the bench-top libraries from individual replicates, from pooled triplicate replicates, and at different average coverage levels. With the AF threshold value at 0.82 (red circles), there is no meaningful difference between microfluidic and bench-top libraries, or between single replicate and pooled triplicates at equal coverage. Due to differences in gene content and genomic structure of the reference and the subject P01 strain, 7% of the reference genome had no coverage in this analysis and 1.9% of the remaining sequence was masked due to poor average read mapping quality (MQ<45; Supplementary Note 9). (**e**) Homology tree constructed based on the number of inter-subject SNPs. (**f**) Heat map of SNPs between each sample, grouped by the patient subject. Asterisks indicate control plate isolates that were expanded from single colonies. Numbers represent median number of SNPs in each subject pair block.

To test the potential for our approach to address the sample-processing bottleneck that limits application of microbial WGS analysis, we applied our system to process libraries from 124*P. aeruginosa* clinical isolates obtained from six randomly selected subjects from Brigham and Women's Hospital (Boston, MA). A total of 12 to 24*P. aeruginosa* colonies were isolated from each subject sample (Fig. 2a; Supplementary Data 3). To empirically determine the single-nucleotide polymorphism (SNP) false detection rate, colonies from two of the six subjects (P03 and P04) were each sampled from ‘control' plates representing expansions of individual primary colonies. We extracted genomic DNA from each culture, normalized the DNA concentrations and loaded the samples into our microfluidic device for library construction. To enable analysis of any errors that occurred during sample preparation, sequencing or analysis^38^, we prepared triplicate technical replicate libraries from each DNA sample (Supplementary Note 6). A single pool of 384 barcoded *P. aeruginosa* and control libraries was sequenced across two HiSeq 2500 lanes (Supplementary Note 7 and 8).

### Low-input libraries support high-specification SNP calling

In order to determine the genomic diversity across isolates and reliably detect sequence variants with important functional consequences like antibiotic resistance, sequencing libraries must not introduce systematic errors and maintain uniform genomic coverage. To compare our microfluidic *Pseudomonas* libraries to those produced using bench-top methods, we instantiated an informatics pipeline for SNP calling (Supplementary Fig. 10) with an allelic fraction (AF) threshold as the primary adjustable parameter controlling sensitivity and specificity (Supplementary Fig. 11). We define the AF as the fraction of quality-adjusted^39^ variant base counts at a given reference position. A lower AF threshold increases SNP calling sensitivity but increases false-positive calls arising from errors that might occur in library construction, sequencing and read mapping operations. We found that setting the AF threshold to 0.82 limited the number of discordant SNPs detected across replicate libraries to zero (using discordant SNPs as a heuristic for erroneous variant calls^38^; Fig. 2b,c). To benchmark our parameterized pipeline, we analysed data from a previous study of SNPs in clinical *Burkholderia dolosa* isolates^40^ and successfully detected all the reported SNPs (Supplementary Data 4).

To validate the actual sensitivity and specificity of SNP calls in analysis of low-input microfluidic sequence libraries versus bench-top libraries, we prepared several libraries from the same isolate sample: three low-input (50 pg) microfluidic libraries and three high-input (24 ng) bench-top libraries, each sequenced to 50 × coverage; and an additional high-input ‘gold standard' bench-top library sequenced to 340 × coverage. At 50 × mean coverage of the 50 pg microfluidic libraries, no false-positive SNPs were detected versus the 340 × high-input library, indicating that accuracy was better than 1.4 × 10^−7^ when calling more than 99% of reference positions. At equal mean coverage, there was no significant decrease in the quality of SNP calls made from the picogram-input libraries produced in our device versus the nanogram-input bench-top libraries (Fig. 2d). In fact, the 50 × low-input consensus base calling accuracy from the 50 pg input samples compares favourably to that reported for advanced single-strand consensus sequencing approaches that depend on extraordinarily deep sequence coverage and extra sample preparation steps (10^−5^; ref. 41). Although we expect replicate library sequencing to suppress rare sample preparation artifacts like PCR errors, our comparison revealed no significant difference in the accuracy of replicate library sequencing compared with single-library sequencing at equivalent depth in our samples (Fig. 2d), indicating that artifacts from library construction affected variant calling at a frequency below 10^−7^.

### Comparative genomics of clinical *Pseudomonas* isolates

In examining sequence variation between isolates, we identified 25,000–90,000 SNP sites across isolates from different subjects (Fig. 2e), but isolates from the same subject were essentially clonal (Fig. 2f; Supplementary Data 5). We detected no SNP sites among three subjects' isolates (P01, P02 and P06) and one of the control plates (P03). Among isolates from subject P05, just two SNP sites were identified, and only one SNP site was identified among isolates from control plate P04, with 2 of 24 isolates showing evidence for the same variant at this locus (Supplementary Note 9). Because the variant base in these two samples was reproducibly detected across the technical replicates in both affected subject P04 isolates (six sequence libraries in total), we can exclude sample preparation and sequencing errors as explanations for the variant calls. Rather, these calls likely arose from a mixture of strains being transferred to the control plate from overlapping colonies on the primary plate or a mutation that occurred during subsequent laboratory culture.

**Figure 3:**
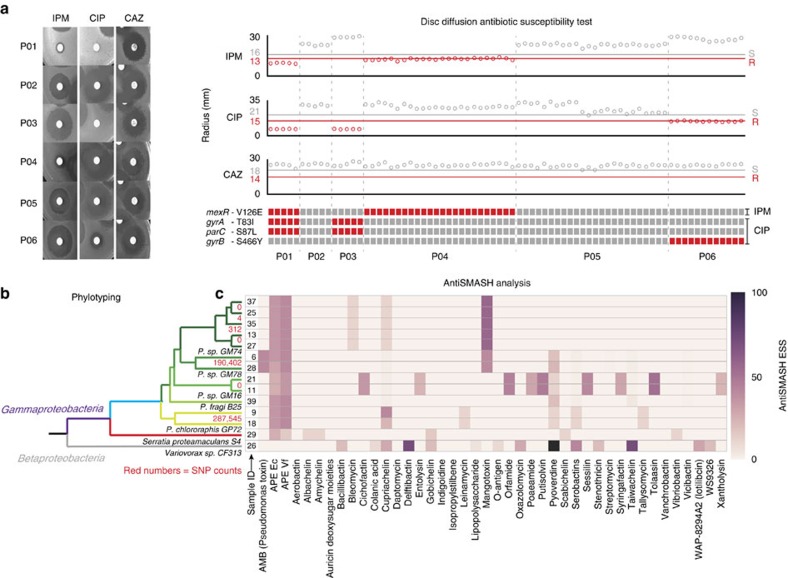
Functional genomics from low-input microbial samples. (**a**) Antibiotic susceptibility phenotyping and genotyping. Antibiotic susceptibility was tested on a randomly sampled subset of isolates from each subject by the disc diffusion susceptibility assay. The drugs tested are IPM, CIP and CAZ (10 μg imipenem, 5 μg ciprofloxacin and 30 μg ceftazidime, respectively). Raw images of one plate from each subject that was analysed (left). Plot shows inhibition zone radius for each antibiotic and known antibiotic resistance SNPs detected by WGS, grouped by subjects (right). The samples with smaller radii close to the Clinical & Laboratory Standards Institute (CLSI) break point (red line) indicate samples that are resistant to the specific antibiotic (grey points). Radii greater than the green line indicates samples identified as susceptible by the CLSI break point (blue points). The first ten samples from subject P05 were measured on a separate day from the remaining P05 samples; the apparent difference in susceptibility in these samples and the remaining P05 samples is most likely due to a systematic difference in the assay (possibly image contrast) on the second day or degradation of the drug sample used between the two measurement sets (we classify all the P05 isolates tested as susceptible to ciprofloxacin). (**b**) Phylotyping of the soil micro-colonies and (**c**) secondary metabolite class prediction using AntiSMASH analysis of *de novo* assembled genomic contigs. The clustering of samples in the phylotyping results is reflected in the secondary metabolite predictions. The red numbers in (**b**) indicate the number of SNPs between the strains spanning each value. The heat map values in (**c**) represent empirical similarity scores^11^.

Our antibiotic resistance phenotype testing and gene content analysis likewise showed homogeneity across isolates from a given subject and extensive differences between isolates from different subjects (Fig. 3a and Supplementary Fig. 12; Supplementary Note 10). The core genes that were common in isolates between subjects were enriched for function in central dogma processes, while genes that varied between subjects were enriched for functions related to DNA transposition, recombination, restriction-modification, and transition metal response genes tied to mercury resistance (Supplementary Fig. 13; Supplementary Data 6 and 7; Supplementary Note 11). To fully validate the small number of variant calls made among the patient-specific isolate populations, the no-calls we made at loci with metrics near our analysis thresholds (Supplementary Fig. 14; Supplementary Data 5; Supplementary Note 9), and the variants responsible for drug resistance (described below), we performed Sanger sequencing, finding agreement with the short-read analysis in all cases.

### WGS sequencing accurately predicts antibiotic resistance

Our sequence analysis of the clinical *Pseudomonas* isolates identified variant bases known to confer resistance to imipenem, ciprofloxacin and ceftazidime, three antibiotics commonly used to treat pulmonary *P. aeruginosa* infections (Fig. 3a; Supplementary Data 8). All 24 isolates from subjects P01 and P04 had a non-synonymous SNP, V126E, in the *mexR* (efflux pump) gene consistent with previously reported imipenem resistance mutations^42^. Similarly, all isolates from subjects P01 and P03 had two SNPs, T83I in the *gyrA* (gyrase A) gene and S87L in the *parC* (topoisomerase IV) gene, each reported to yield ciprofloxacin resistance^43^. All 12 isolates from subject P06 had a ciprofloxacin resistance mutation, S466Y in the *gyrB*^44^ (gyrase B) gene, that was not found in the other subjects. In our drug resistance phenotype test^45^ (Fig. 3a; Supplementary Note 10), 100% of the phenotypically observed variability in resistance was explained by our low input, high-throughput WGS data (Fig. 3a).

### Direct *de novo* WGS analysis of soil micro-colonies

Our microfluidic platform showed data performance equal to bench-top library construction methods with orders of magnitude less input, lending the confidence to carry out strain and natural product prediction from the iChip micro-colony samples. We *de novo* assembled the genome of each micro-colony data set produced as described above (Supplementary Data 9) and phylotyped the micro-colonies at the strain level using a multi-locus sequence typing analysis^46^.

Of the 14 colonies analysed, 12 represented five different known *Pseudomonas* strains (*P. sp* GM74, *P. sp* GM78, *P. sp* GM16, *P. fragi* B25 and *P. chlororaphis* GP72). We identified the remaining two colonies as Serratia proteamaculans S4 and Varivorax sp. CF313, respectively (Fig. 3b and Supplementary Fig. 15). Our high-GC *Pseudomonas* assemblies, based on low-input sample-processing direct from cells, were more contiguous than recently reported draft environmental Pseudomonas assemblies produced from sequence libraries prepared by standard, high-input methods^47^ (Supplementary Data 9). The device was also more reliable in producing libraries from our micro-colony samples, as two of the bench-top libraries dropped out entirely during the sample preparation process (Fig. 1f and Supplementary Fig. 16) and six of the fourteen bench-top libraries were at or below a library complexity of 200 genomic equivalents, the minimum required to enable 50 × unique coverage with acceptable read duplication rate.

Soil samples are frequently mined for natural products, recently yielding teixobactin, a promising agent under development for treating Gram-positive bacterial infections^48^. We analysed our micro-colony draft genomes for natural product genes using the anti-SMASH tool^11^ (Fig. 3c). While the metabolite-producing gene cluster profiles between the device libraries and bench-top libraries mostly agreed (except where the bench-top samples dropped out entirely), the *Serratia* and *Varivorax* samples gave divergent results, with the higher-quality microfluidic libraries likely producing more accurate gene calls (Supplementary Fig. 16).

## Discussion

Despite the ability of Illumina's HiSeq and NextSeq platforms to process hundreds of microbial sequence libraries per run, genomic analysis is underutilized in epidemiology, clinical care and natural product discovery due to the complexity, limited throughput, labour-intensity, and input quantity requirements of available sample preparation methods. Here, we piloted an automated microfluidic system that integrates at high throughput all the key steps in NGS sample preparation: cell concentration, lysis, fragmentation, adaptor tagging, fragment purification and size selection (Supplementary Fig. 2; Supplementary Data 2, 10 and 11). By lowering input requirements 200-fold, our system also enables new applications such as rapid analysis of slow-growing pathogen isolates and direct analyses of samples with limited biomass.

We constructed nearly 400 WGS sequence libraries from 124 isolates of *P. aeruginosa* collected from six patients. Our analysis revealed tremendous inter-subject diversity in gene content and genome sequence even though all six samples were collected from the same hospital within a period of a few weeks. Despite the dynamic accessory genome known in *Pseudomonas*, we found negligible intra-subject variation in our samples, indicating that the infected sites contained essentially clonal *Pseudomonas* populations, not the diverse *Pseudomonas* populations often described in the chronic pulmonary infections common among cystic fibrosis patients (Supplementary Note 12). The population signature we observed is consistent with community-acquired infection or infection of these patients from their own microbiota in the hospital setting.

The genomic determinants of imipenem and ciprofloxacin resistance were apparent in our variant calls and concordant with phenotypic susceptibility assays. We also found substantial gene content and sequence variation across strains with the same multiple resistance phenotype, indicating that these strains most likely acquired resistance to the same set of drugs independently. As is often found in *Pseudomonas*, we noticed diversity in the gene content across the isolates from different patients, notably enrichment of DNA integration and restriction functions in the variable genome that may be related to horizontal gene transfer, subject-specific prophage (Supplementary Figs 17 and 18) and other putatively exogenous sequences (Supplementary Fig. 19; Supplementary Data 12; Supplementary Note 13), as well as mercury resistance elements, which have previously been linked to the development of antibiotic resistance^49^,^50^.

These analyses were enabled by the Q68+ SNP calling performance achieved by the tuned variant caller^38^. Replicate sequencing that enabled this performance increases the need for automation of sample preparation and low-input sample preparation, but has the power to discriminate sequencing errors from errors introduced during library construction including base damage, PCR-derived mutations, and chimeras.

We demonstrated that the microfluidic system's integrated DNA extraction capability works at very low-input levels for challenging sample types like *M. tuberculosis* cells and soil micro-colonies. This eliminates the need for pre-amplification by whole-genome amplification, which increases workflow complexity and degrades data quality. The input reduction to thousands of cells could cut diagnostic test times in half for slow-growing pathogens like *M. tuberculosis*. Lowering input requirements also enables new organism and natural product discovery by direct WGS analysis of environmental microbes that are hard to culture, such as the iChip micro-colonies. Side-by-side comparison of soil micro-colony WGS analysis with an optimized bench-top procedure makes clear the superior performance of the new microfluidic method in reliability and critical data metrics. Future synthetic biology approaches^51^ to synthesize natural product genes for expression in industrial production strains would place even higher demands on sequence and assembly quality. Re-discovery of known natural products is a limiting factor in the discovery of novel bioactive compounds^52^, placing a premium on isolating the most challenging-to-grow organisms that are enriched in diversity versus known microbes.

We expect the increased automation, throughput and low-input capability of our microfluidic library construction method to enable a wide variety of future applications. Current library preparation protocols require nanograms to micrograms of DNA that have been previously extracted from cells. Some protocols require that DNA be pre-fragmented and size-selected in addition. Eliminating such challenging input requirements could expand the use of less invasive but low-yield sampling and biopsy procedures, enable direct pathogen identification in tiny microbiome samples, better enrichment of cell types or tissue regions of interest in clinical micro-samples, and eliminate whole-genome pre-amplification from some workflows^53^. The high throughput and high accuracy sample preparation method presented here will power applications including precision medicine, genomic surveillance, antibiotic resistance tracking^2^,^3^,^4^,^5^ and novel organism/natural product discovery on large scales.

## Methods

### Microfluidic device fabrication

Microfluidic devices were fabricated by multi-layer soft lithography of PDMS, a transparent silicone elastomer, on a mould comprised of a silicon wafer patterned with photoresist. Mould and device fabrication were carried out at the Broad Institute. Separate moulds were used to cast a control layer of height 50 μm and a flow layer of height 6 mm. The two layers were partially cured, aligned manually and thermally bonded by further curing. Inlet ports were punched and the two-layer PDMS device was bonded to a glass slide after activation by air plasma exposure.

The flow layer mould was patterned in five steps: (1) rectangular 15 μm, (2) rectangular 5 μm, (3) rounded 15 μm, (4) rectangular 30 μm and (5) rectangular 100 μm features. The rectangular features were made by spin coating SU-8 2001 (5 μm), 2015 (15 and 30 μm) and 2050 (100 μm) photoresists (Microchem) on a silicon wafer. The coated wafers were patterned by ultraviolet exposure (OAI 206 mask aligner) through a mask printed at 20,000 dpi (Fineline; see Autocad design files for each layer in supplementary material), followed by feature development in SU-8 developer (Microchem). The rounded features were produced by spin coating AZ-4620 photoresist (Microchem) after coating with Hexamethyldisilazane (Sigma) and air-drying, patterning the wafer with UV exposure and a mask, developing in the AZ 400 K developer (Microchem), and slowly heating (from 65 C to 190 C over 4 h) the wafer above the resist Tg (120 C) to round the features. The control layer mould was patterned in two steps: (1) rectangular 15 μm and (2) rectangular 90 μm features, using methods similar to those for the flow layer.

Device production utilized standard soft lithography protocols. PDMS (Momentive) was mixed at 5:1 silicone to cross-linker ratio in a Thinky AR250 mixer, poured onto the flow layer mould, degassed in a vacuum chamber and cured by baking for 1 h at 80 °C. PDMS at a 20:1 silicone to cross-linker ratio was spin coated on the valve layer mould to a height of 50 μm, then baked at 80 °C for 40 min. The two layers were aligned under a stereomicroscope (Nikon), and further baked for 2 h at 80 °C to complete thermal bonding. Inlet holes were punched into the two-layered PDMS device (Syneo, ID 660 μm tips) on the designated input/output port features. The device was then exposed to atmospheric plasma for 30 s at a pressure of 1.3 mbar (Diener ATTO), bonded to a clean glass slide and baked for 3 h at 80 °C.

### Device design file and controller

Flow and control layers were pneumatically activated by an array of 40 solenoid valves (Pneumadyne) controlled by a USB interface (McMaster) connected to a computer. The device controller was home-built following designs and procedures specified by Dr Rafael Gómez-Sjöberg and the Prof. Stephen Quake group at Stanford University as described at: https://sites.google.com/site/rafaelsmicrofluidicspage/valve-controllers/usb-based-controller.

The valve operation pressure was 40 PSI and sample/reagent solutions were driven at 20 PSI.

The device CAD file is publicly available on: https://sourceforge.net/projects/sk-dev-cad-analysis-software/files/Kim_supplementaryfiles.zip /download.

### Clinical *P*. aeruginosa isolate sample collection and culture

The clinical samples used in this study were discards from the Brigham and Women's Hospital microbiology lab, and were disconnected from patient meta-data. Samples from selected sites of infection were streaked on selective media plates containing MacConkey agar. 12 to 24 *P. aeruginosa* colonies were identified by appearance and randomly picked (Fig. 2a). *P. aeruginosa* isolates were received at the Broad Institute as frozen liquid cultures. They were grown to mid-log phase at a concentration of 5 × 10^8^ cells ml^−1^ in lysogeny broth (LB). Culture density was monitored by OD600 using a UV–vis spectrophotometer.

### DNA extraction from clinical isolates (bench-top method)

DNA was purified from bacterial cultures using Qiagen DNeasy Blood and Tissue kits on the QIAcube instrument (Qiagen). The cell input was between one and two million cells per sample. After extraction, the concentration of purified gDNA was measured by absorption at 260 nm (Nanodrop) and normalized to 20 ng μl.

### *M. tuberculosis* culture

Two *M. tuberculosis* clinical isolates (OFXR14 and OFXR16) were grown in Middlebrook 7H9 medium supplemented with OADC (Becton Dickinson), 0.05% Tween 80 (Sigma) and 0.2% glycerol at 37C with shaking. Samples were taken at OD600 0.05 to 0.1, which corresponds to ∼1.5–3 × 10^7^ bacterial cells per ml. A 100 μl culture sample from each isolate was heat inactivated at 80 °C for 2 h, flash frozen and used for all analyses.

### iChip soil bacteria culture

A 1 g sample of soil collected from a private garden in Boston, MA was agitated vigorously for 10 min in 10 ml sterile phosphate buffered saline (PBS). The soil was left to settle for 5 min and the supernatant was diluted again in PBS. Dilutions were then mixed with molten agar media (0.1 g starch, 1.0 g casamino acids, 15 g technical agar per 1 l of H_2_O). The soil suspension was diluted further with agar to achieve an average concentration of one cell per 100 μl. Then 100 μl of the soil-agar suspension was dispensed into the each well of an iChip^22^, which had a 0.03 μm polycarbonate membrane attached to the bottom via silicone glue. After the agar suspension was solid, a 0.03 μm polycarbonate membrane was attached to the top of the iChip using silicone glue. The iChip was incubated in direct contact with moist soil in the dark for 2 weeks. After 2 weeks, iChips were disassembled and individual colonies were picked with sterile toothpicks using a dissection microscope, and placed into 15 μl PBS for further analysis.

### Tagmentation (bench-top method, 24 ng input)

Bench-top library construction was done following the Illumina Nextera protocol for tagmentation. 5 μl of 4.8 ng μl^−1^ (24 ng total) of purified gDNA was mixed with 15 μl of Nextera enzyme (Illumina), 5 μl of 5 × Tagmentation buffer (50 mM Tris-HCl pH 8.0 (Sigma), 20 mM MgCl_2_ (Sigma)) and 10 μl of H_2_O, then incubated for 10 min at 58 °C. 2.5 μl of stop solution (2.5% wt per vol SDS (Sigma) in H_2_O) was added to the mixture, followed by incubation for 10 min at 72 °C. Tagmented DNA was purified by mixing with 49.5 μl SPRI bead suspension (Beckman-Coulter), binding for 10 min at 25 °C, magnetically separating the beads, washing twice with ethanol and eluting the product off the beads with 50 μl of 10 mM Tris-HCl pH 8.0.

### Commercial DNA extraction method (bench-top bead beating)

We attempted using the MoBio Power-soil kit (Qiagen) with 7 μl of cells (∼10^4^ total cells) to extract gDNA for library construction but did not obtain a sufficient quantity of tagmented DNA fragments after library construction (<1 picograms of amplifiable tagmented product; data not shown).

### Library quantification (qPCR)

Before enrichment PCR, an aliquot from each library produced on the device was quantified by qPCR to verify successful library construction. qPCR was performed by mixing 1 μl of tagmented product with 0.5 μl Eva green dye (Evrogen), 0.5 μl of Rox reference dye (ROCHE), 0.5 μl of N12 Nextera barcoding primer (Illumina), 0.5 μl of E502 Nextera barcoding primer (Illumina), 4 μl of DNA polymerase ready mix (Illumina) and 3 μl of H_2_O, then performing qPCR in a real-time thermocycler (Stratagene MX3005p). The thermal programme was: 5 min at 72 °C, 1 min at 95 °C, then 40 cycles of (1) 10 s at 95 °C, (2) 30 s at 60 °C and (3) 30 s at 72 °C. To quantify the properly adapted tagmented library molecules in each sample the qPCR amplification curve of each sample was compared with the curves resulting from analysis of purified standards (amplified, size-selected library) of similar average molecular weight and GC content. The reference libraries were quantified using the Qubit method (ThermoFisher) and Kapa library quantification kits (Kapa Biosystems).

### Enrichment/barcoding PCR and sample pooling

The sequencing libraries created on the microfluidic devices were barcoded using the Broad Institute's dual barcoding primers (Broad Genomics Platform), for which validation was not yet complete. Eight libraries were lost in the enrichment PCR step from the *Pseuodomonas* set due to bad primers.

4 μl of each tagmented sample library (∼5 pg) was mixed with 0.5 μl of primer 1, 0.5 μl of primer 2, 4 μl of amplification mix (Illumina) and 1 μl of H_2_O. Our libraries were amplified by 15 cycles of PCR (optimized for 5 pg input), SPRI purified and Qubit quantified. The libraries were then pooled into a single mix in equal concentrations based on their Qubit-measured concentrations.

### DNA sequencing

Quality assessment runs (library construction efficiency, sensitivity versus specificity, k-mer abundance and fragment size distribution) and production runs of test libraries were carried out by sequencing across variably configured Illumina MiSeq runs (2 × 50, 2 × 75, 2 × 150, 2 × 250 and 2 × 300). The quality assessed samples were sequenced on the Illumina Hiseq 2500 platform (2 × 125 cycles or 2 × 101 cycles) in the Broad Genomics Platform.

### Antibiotic susceptibility testing

Clinical *Pseudomonas aeruginosa* isolates were grown to mid-log phase in Luria broth (LB), and 100 μl of each culture was spread on an LB agar plate and allowed to dry for 15 min face-up at room temperature. Disks impregnated with ceftazidime (30 μg, Becton Dickinson), ciprofloxacin (5 μg, Becton Dickinson) or imipenem (10 μg, Becton Dickinson) were placed at evenly spaced distances on these plates, which were then incubated for 16 h at 37 °C. Plates were imaged using a FluorChem FC2 system (Alpha-Innotech) set to the visible range, and the radius of the zone of inhibition around each disk was measured using a custom Matlab image analysis script. These radii were compared with standards determined by the Clinical Laboratory Standards Institute (CLSI). The breakpoints for each antibiotic are as follow (*S* and *R* stand for susceptible and resistant, respectively):

1. Ciprofloxacin: S>21 mm; *R*<15 mm
2. Imipenem: *S*>16 mm; *R*<13 mm
3. Ceftazidime: *S*>18 mm; *R*<14 mm

### Analysis software and commands

Software and commands used for sequencing analysis are available in the supplementary methods. Custom analysis software is publicly available on https://sourceforge.net/projects/sk-dev-cad-analysis-software/files/Kim_supplementaryfiles.zip/download.

### Data availability

#### Primary accessions

National Center for Biotechnology Information (NCBI) Sequence Read Archive (SRA)

clinical *P. aeruginosa* raw reads    fastq PRJNA295070

clinical *P. aeruginosa de novo* assemblies fasta PRJNA295070

low-input *E. coli* raw reads fastq PRJNA295070

*M. tuberculosis* raw reads fastq PRJNA295070

soil micro-colonies raw reads fastq PRJNA295070

#### Referenced accessions

NCBI Reference sequence

*Pseudomonas aeruginosa* UCBPP-PA14 complete genome NC_008463.1

*Mycobacterium tuberculosis* OFXR-14 scaffold GCF_000660185.1

*Mycobacterium tuberculosis* OFXR-16 scaffold GCF_000660225.1

## Additional information

**How to cite this article:** Kim, S. *et al*. High-throughput automated microfluidic sample preparation for accurate microbial genomics. *Nat. Commun.* **8,** 13919 doi: 10.1038/ncomms13919 (2017).

**Publisher's note:** Springer Nature remains neutral with regard to jurisdictional claims in published maps and institutional affiliations.

## Supplementary Material

Supplementary Information Supplementary Figures, Supplementary Notes, Supplementary Methods, and Supplementary References.

Supplementary Data 1 Cost comparison different WGS sample preparation technologies for Illumina NGS. The listed cost includes labor and consumables.

Supplementary Data 2 List of samples. The device library construction efficiencies and resulting library fragment size distribution (supplementary note 4) agreed well with our input estimates. The clinical Pseudomonas genomic DNA was extracted using the Qiagen Blood & Tissue kit. The cell input samples were lysed by a combination of heat, detergents, and our own mixture of hydrolytic enzymes (Methods).

Supplementary Data 3 Clinical sample collection methods and isolate counts by subject. Secondary plates are re-streaks from a single primary colony and served as technical controls.

Supplementary Data 4 SNP detection cross-validation. All mutations in Lieberman et al. (2014) were identified using our analysis pipeline. We downloaded the raw Lieberman et al. (2014) fastq files deposited on NCBI and ran our analysis pipeline with the coverage, mapping, and allelic fraction filtering threshold set to 6, 45, and 0.82, respectively, the same filtering thresholds used in our clinical Pseudomonas aeruginosa study. We identified 186/187 SNPs reported in Lieberman et al. (2014) plus ten more SNPs. All 187 SNPs were identified by lowering our our coverage threshold to 4, but resulted in additional SNP calls.

Supplementary Data 5 Table of mutations found in each isolate. The numbers in each colony box indicate the allelic fraction. The red and green fill indicate are mutations that passed the filtering threshold (coverage > 6, mapping quality > 45, and allelic fraction > 82), while the clear boxes represent "no calls" that were masked because they did not pass the filtering threshold. Mutations are categorized as synonymous, non-synonymous, and intergenic. AF values for each SNP ranges from 0 to 1, where 0 and 1 represent pure allelic sets while intermediate values indicate loci where minor allelic bases are present (supplementary note 9).

Supplementary Data 6 Gene set enrichment analysis of the variable region of our Pseudomonas aeruginosa Isolates. Functional categories with different representation in P. aeruginosa variable and core genomes. Second and third column show the number of genes mapping to the category in question in core and variable genomes, respectively. Fourth and fifth column show the fraction of annotated genes mapping to the given category. Only functional categories with false discovery rate corrected *p*-value < 0.2 are shown. This classification was done with the best-annotated genes according to Gene Ontology (GO) categories and UniRef50.

Supplementary Data 7 Details of genes in the two categories, GO:1990267 and GO:0010038 (response to transition metal nanoparticle and response to metal ion, respectively). 10/11 genes in these categories are response genes to mercury reported to result in mercury resistance.

Supplementary Data 8 List of resistance mutations. Resistance mutations were found in subjects 1, 3, 4, and 5 for imipenem (IPM) or ciprofloxacin (CIP). All isolates from each patient had identical resistance mutations and phenotypic resistance to the antibiotics.

Supplementary Data 9 Table of de novo assembly statistics of our low-input (~10^4 cells) soil microcolonies processed using our device. Our Pseudomonas assemblies were more contiguous than recently reported draft environmental Pseudomonas assemblies produced from sequence libraries prepared by standard, high-input methods. The draft assemblies compared are from Winsor, G. L. et al. Nucleic Acids Res. 44, D646-653 (2015).

Supplementary Data 10 WGS library construction cost survey. Library construction costs on average $269 dollars per sample at these sequencing centres.

Supplementary Data 11 Cost estimate for microfluidic WGS sample preparation. The cost here is a direct cost that includes realistic technician labor, pipet tips, and the reagent cost, but excludes institutional facilities and administrative expenses and any profit margin or recoupment of development expenses.

Supplementary Data 12 Putative sites of horizontally transferred sequences. Table of blast results showing contigs from patient isolates containing DNA sequences with extensive homology to multiple database hits (Pseudomonas aeruginosa PA14, other bacterial, and Pseudomonas phage genomes). BlastN was used to identify homologous regions in de novo assembled contigs. Pseudomonas aeruginosa host gene, identified by BlastX with 100% identity, for contigs with both viral and bacterial sequences are shown in parentesis. Strains from P01, P02, and P06 both showed sequence highly complementary to a ~10kb segment of Alicycliphilus denitrificans BC containing several genes associated with genetic mobility (ALIDE_RS19890, "integrating conjugative element protein"; ALIDE_RS19895, "integrase"; ALIDE_RS19900, "single-stranded DNA-binding protein"), and the P03 strain has a ~5kb region homologous to the same Alicyliphilus strain that encodes a predicted helicase (ALIDE_RS19940).

Supplementary Data 13 Table of de novo assembly statistics of clinical pseudomonas aeruginosa isolates processed using our device. The input was 50 pg of purified genomic DNA, and each isolate was sequenced to about 50x mean coverage.

## References

[b1] Yatsunenko T. . Human gut microbiome viewed across age and geography. Nature 486, 222–227 (2012). 22699611 10.1038/nature11053 PMC3376388

[b2] Marvig R. L., Sommer L. M., Molin S. & Johansen H. K. Convergent evolution and adaptation of *Pseudomonas aeruginosa* within patients with cystic fibrosis. Nat. Genet. 47, 57–64 (2014). 25401299 10.1038/ng.3148

[b3] Dallman T. J. . Whole-genome sequencing for national surveillance of shiga toxin-producing *Escherichia coli* O157. Clin. Infect. Dis. 61, 305–312 (2015). 25888672 10.1093/cid/civ318 PMC4542925

[b4] Nasser W. . Evolutionary pathway to increased virulence and epidemic group A Streptococcus disease derived from 3,615 genome sequences. Proc. Natl Acad. Sci. USA 111, E1768–E1776 (2014). 24733896 10.1073/pnas.1403138111 PMC4035937

[b5] Chewapreecha C. . Dense genomic sampling identifies highways of pneumococcal recombination. Nat. Genet. 46, 305–309 (2014). 24509479 10.1038/ng.2895 PMC3970364

[b6] Smith E. E. . Genetic adaptation by *Pseudomonas aeruginosa* to the airways of cystic fibrosis patients. Proc. Natl Acad. Sci. USA 103, 8487–8492 (2006). 16687478 10.1073/pnas.0602138103 PMC1482519

[b7] Snitkin E. S. . Tracking a hospital outbreak of carbapenem-resistant Klebsiella pneumoniae with whole-genome sequencing. Sci. Transl. Med. 4, 148ra116 (2012). 10.1126/scitranslmed.3004129 PMC3521604 22914622

[b8] Centers for Disease Control and Prevention, Antibiotic-Resistant Gonorrhea - STD information from CDC. Available at https://www.cdc.gov/std/gonorrhea/arg/basic.htm.

[b9] Obama B. Executive Order--Combating Antibiotic-Resistant Bacteria | whitehouse.gov. the White house (2014). Available at https://www.whitehouse.gov/the-press-office/2014/09/18/executive-order-combating-antibiotic-resistant-bacteria>.

[b10] Newman D. J. & Cragg G. M. Natural products as sources of new drugs over the 30 years from 1981 to 2010. J. Nat. Prod. 75, 311–335 (2012). 22316239 10.1021/np200906s PMC3721181

[b11] Weber T. . antiSMASH 3.0--a comprehensive resource for the genome mining of biosynthetic gene clusters. Nucleic Acids Res. 43, W237–W243 (2015). 25948579 10.1093/nar/gkv437 PMC4489286

[b12] Kim H. . A microfluidic DNA library preparation platform for next-generation sequencing. PLoS ONE 8, e68988 (2013). 23894387 10.1371/journal.pone.0068988 PMC3718812

[b13] de Bourcy C. F. A. . A quantitative comparison of single-cell whole genome amplification methods. PLoS ONE 9, e105585 (2014). 25136831 10.1371/journal.pone.0105585 PMC4138190

[b14] Kram K. E. & Finkel S. E. Rich medium composition affects *Escherichia coli* survival, glycation, and mutation frequency during long-term batch culture. Appl. Environ. Microbiol. 81, 4442–4450 (2015). 25911475 10.1128/AEM.00722-15 PMC4475895

[b15] Blainey P. C. The future is now: single-cell genomics of bacteria and archaea. FEMS Microbiol. Rev. 37, 407–427 (2013). 23298390 10.1111/1574-6976.12015 PMC3878092

[b16] Salter S. J. . Reagent and laboratory contamination can critically impact sequence-based microbiome analyses. BMC Biol. 12, 87 (2014). 25387460 10.1186/s12915-014-0087-z PMC4228153

[b17] Jones M. B. . Library preparation methodology can influence genomic and functional predictions in human microbiome research. Proc. Natl Acad. Sci. USA 112, 14024–14029 (2015). 26512100 10.1073/pnas.1519288112 PMC4653211

[b18] Brooks J. P. . The truth about metagenomics: quantifying and counteracting bias in 16S rRNA studies. BMC Microbiol. 15, 66 (2015). 25880246 10.1186/s12866-015-0351-6 PMC4433096

[b19] Witney A. A. . Clinical use of whole genome sequencing for *Mycobacterium tuberculosis*. BMC Med. 14, 46 (2016). 27004841 10.1186/s12916-016-0598-2 PMC4804576

[b20] Wilson M. R. . Actionable diagnosis of neuroleptospirosis by next-generation sequencing. N. Engl. J. Med. 370, 2408–2417 (2014). 24896819 10.1056/NEJMoa1401268 PMC4134948

[b21] Naccache S. N. . A cloud-compatible bioinformatics pipeline for ultrarapid pathogen identification from next-generation sequencing of clinical samples. Genome Res. 24, 1180–1192 (2014). 24899342 10.1101/gr.171934.113 PMC4079973

[b22] Nichols D. . Use of ichip for high-throughput in situ cultivation of &quot;uncultivable&quot; microbial species. Appl. Environ. Microbiol. 76, 2445–2450 (2010). 20173072 10.1128/AEM.01754-09 PMC2849220

[b23] Anderson S. Shotgun DNA sequencing using cloned DNase I-generated fragments. Nucleic Acids Res. 9, 3015–3027 (1981). 6269069 10.1093/nar/9.13.3015 PMC327328

[b24] Ramsköld D. . Full-length mRNA-Seq from single-cell levels of RNA and individual circulating tumor cells. Nat. Biotechnol. 30, 777–782 (2012). 22820318 10.1038/nbt.2282 PMC3467340

[b25] Buenrostro J. D., Giresi P. G., Zaba L. C., Chang H. Y. & Greenleaf W. J. Transposition of native chromatin for fast and sensitive epigenomic profiling of open chromatin, DNA-binding proteins and nucleosome position. Nat. Methods 10, 1213–1218 (2013). 24097267 10.1038/nmeth.2688 PMC3959825

[b26] Robertson G. . Genome-wide profiles of STAT1 DNA association using chromatin immunoprecipitation and massively parallel sequencing. Nat. Methods 4, 651–657 (2007). 17558387 10.1038/nmeth1068

[b27] Melin J. & Quake S. R. Microfluidic large-scale integration: the evolution of design rules for biological automation. Annu. Rev. Biophys. Biomol. Struct. 36, 213–231 (2007). 17269901 10.1146/annurev.biophys.36.040306.132646

[b28] Morinishi L. S. & Blainey P. Simple bulk readout of digital nucleic acid quantification assays. J. Vis. Exp. e52925 (2015). 10.3791/52925 PMC4692624 26436576

[b29] Caruccio N. Preparation of next-generation sequencing libraries using Nextera technology: simultaneous DNA fragmentation and adaptor tagging by *in vitro* transposition. Methods Mol. Biol. 733, 241–255 (2011). 21431775 10.1007/978-1-61779-089-8_17

[b30] DeAngelis M. M., Wang D. G. & Hawkins T. L. Solid-phase reversible immobilization for the isolation of PCR products. Nucleic Acids Res. 23, 4742–4743 (1995). 8524672 10.1093/nar/23.22.4742 PMC307455

[b31] Kim S. . High-throughput single-molecule optofluidic analysis. Nat. Methods 8, 242–245 (2011). 21297618 10.1038/nmeth.1569 PMC3075913

[b32] Bhattacharyya A. & Klapperich C. M. Thermoplastic microfluidic device for on-chip purification of nucleic acids for disposable diagnostics. Anal. Chem. 78, 788–792 (2006). 16448052 10.1021/ac051449j

[b33] Tan S. J. . A microfluidic device for preparing next generation DNA sequencing libraries and for automating other laboratory protocols that require one or more column chromatography steps. PLoS ONE 8, e64084 (2013). 23894273 10.1371/journal.pone.0064084 PMC3722208

[b34] Balagaddé F. K., You L., Hansen C. L., Arnold F. H. & Quake S. R. Long-term monitoring of bacteria undergoing programmed population control in a microchemostat. Science 309, 137–140 (2005). 15994559 10.1126/science.1109173

[b35] Parkinson N. J. . Preparation of high-quality next-generation sequencing libraries from picogram quantities of target DNA. Genome Res. 22, 125–133 (2012). 22090378 10.1101/gr.124016.111 PMC3246199

[b36] Li H. Mathematical Notes on SAMtools Algorithms. (2010). Available at https://www.broadinstitute.org/gatk/media/docs/Samtools.pdf.

[b37] Kelley D. R., Schatz M. C. & Salzberg S. L. Quake: quality-aware detection and correction of sequencing errors. Genome Biol. 11, R116 (2010). 21114842 10.1186/gb-2010-11-11-r116 PMC3156955

[b38] Robasky K., Lewis N. E. & Church G. M. The role of replicates for error mitigation in next-generation sequencing. Nat. Rev. Genet. 15, 56–62 (2014). 24322726 10.1038/nrg3655 PMC4103745

[b39] Walker B. J. . Pilon: an integrated tool for comprehensive microbial variant detection and genome assembly improvement. PLoS ONE 9, e112963 (2014). 25409509 10.1371/journal.pone.0112963 PMC4237348

[b40] Lieberman T. D. . Genetic variation of a bacterial pathogen within individuals with cystic fibrosis provides a record of selective pressures. Nat. Genet. 46, 82–87 (2014). 24316980 10.1038/ng.2848 PMC3979468

[b41] Schmitt M. W. . Detection of ultra-rare mutations by next-generation sequencing. Proc. Natl Acad. Sci. USA 109, 14508–14513 (2012). 22853953 10.1073/pnas.1208715109 PMC3437896

[b42] Pai H., Kim J., Lee J. H., Choe K. W. & Gotoh N. Carbapenem resistance mechanisms in *Pseudomonas aeruginosa* clinical isolates. Antimicrob. Agents Chemother. 45, 480–484 (2001). 11158744 10.1128/AAC.45.2.480-484.2001 PMC90316

[b43] Lomholt J. A. & Kilian M. Ciprofloxacin susceptibility of *Pseudomonas aeruginosa* isolates from keratitis. Br. J. Ophthalmol. 87, 1238–1240 (2003). 14507757 10.1136/bjo.87.10.1238 PMC1920786

[b44] Bruchmann S., Dötsch A., Nouri B., Chaberny I. F. & Häussler S. Quantitative contributions of target alteration and decreased drug accumulation to *Pseudomonas aeruginosa* fluoroquinolone resistance. Antimicrob. Agents Chemother. 57, 1361–1368 (2013). 23274661 10.1128/AAC.01581-12 PMC3591863

[b45] Bauer A. W., Kirby W. M., Sherris J. C. & Turck M. Antibiotic susceptibility testing by a standardized single disk method. Am. J. Clin. Pathol. 45, 493–496 (1966). 5325707

[b46] Darling A. E. . PhyloSift: phylogenetic analysis of genomes and metagenomes. PeerJ 2, e243 (2014). 24482762 10.7717/peerj.243 PMC3897386

[b47] Winsor G. L. . Enhanced annotations and features for comparing thousands of Pseudomonas genomes in the Pseudomonas genome database. Nucleic Acids Res. 44, D646–D653 (2015). 26578582 10.1093/nar/gkv1227 PMC4702867

[b48] Ling L. L. . A new antibiotic kills pathogens without detectable resistance. Nature 517, 455–459 (2015). 25561178 10.1038/nature14098 PMC7414797

[b49] Skurnik D. . Is exposure to mercury a driving force for the carriage of antibiotic resistance genes? J. Med. Microbiol. 59, 804–807 (2010). 20339018 10.1099/jmm.0.017665-0

[b50] Rowland I. R., Robinson R. D. & Doherty R. A. Effects of diet on mercury metabolism and excretion in mice given methylmercury: role of gut flora. Arch. Environ. Health 39, 401–408 (1984). 6524959 10.1080/00039896.1984.10545872

[b51] Smanski M. J. . Synthetic biology to access and expand nature's chemical diversity. Nat. Rev. Microbiol. 14, 135–149 (2016). 26876034 10.1038/nrmicro.2015.24 PMC5048682

[b52] Baltz R. H. Antimicrobials from actinomycetes: back to the future. Microbe Am. Soc. Microbiol. 2, 125 (2007).

[b53] Fitzsimons M. S. . Nearly finished genomes produced using gel microdroplet culturing reveal substantial intraspecies genomic diversity within the human microbiome. Genome Res. 23, 878–888 (2013). 23493677 10.1101/gr.142208.112 PMC3638143

