# Supplementary material for: High-throughput automated microfluidic sample preparation for accurate microbial genomics
Source: Nat Commun. 2017 Jan 27;8:13919. doi: 10.1038/ncomms13919 (PMC5290157; doi:10.1038/ncomms13919)
Supplement: Supplementary Information — Supplementary Figures, Supplementary Notes, Supplementary Methods, and Supplementary References. [file ncomms13919-s1.pdf]

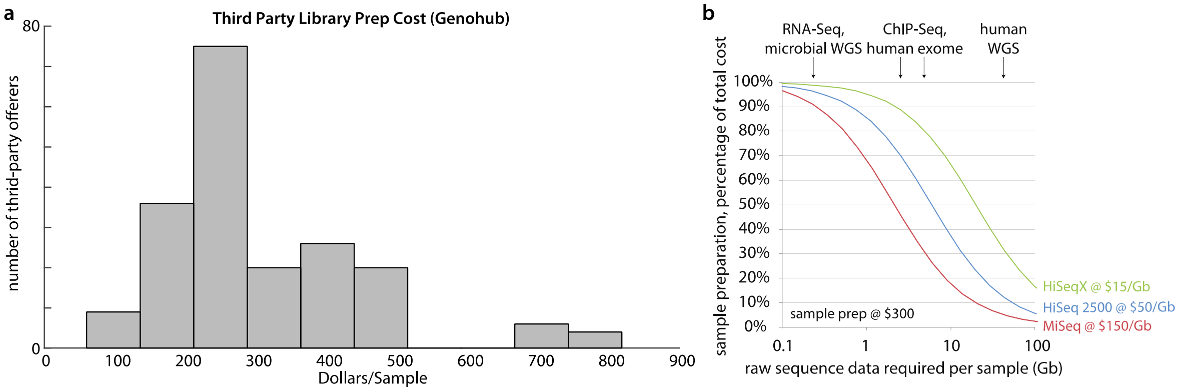

Supplementary Figure 1. Cost of sample preparation as share of total cost for short-read genomic data production. a) Third party library construction cost survey. The library construction costs from 196 third party service providers (academic & non-academic) in the US. This information was obtained from https://genohub.com/ on 12/4/2015. These prices do not include the DNA extraction step, which we estimate adds $50 - $100 to the total sample preparation cost. **b)** Model assumes the costs indicated. More than half of total cost is attributable to sample preparation for genomics applications (listed at top of plot) when HiSeq platforms are used (except human WGS). Microbial WGS is severely bottlenecked by sample preparation costs on MiSeq and HiSeq today.

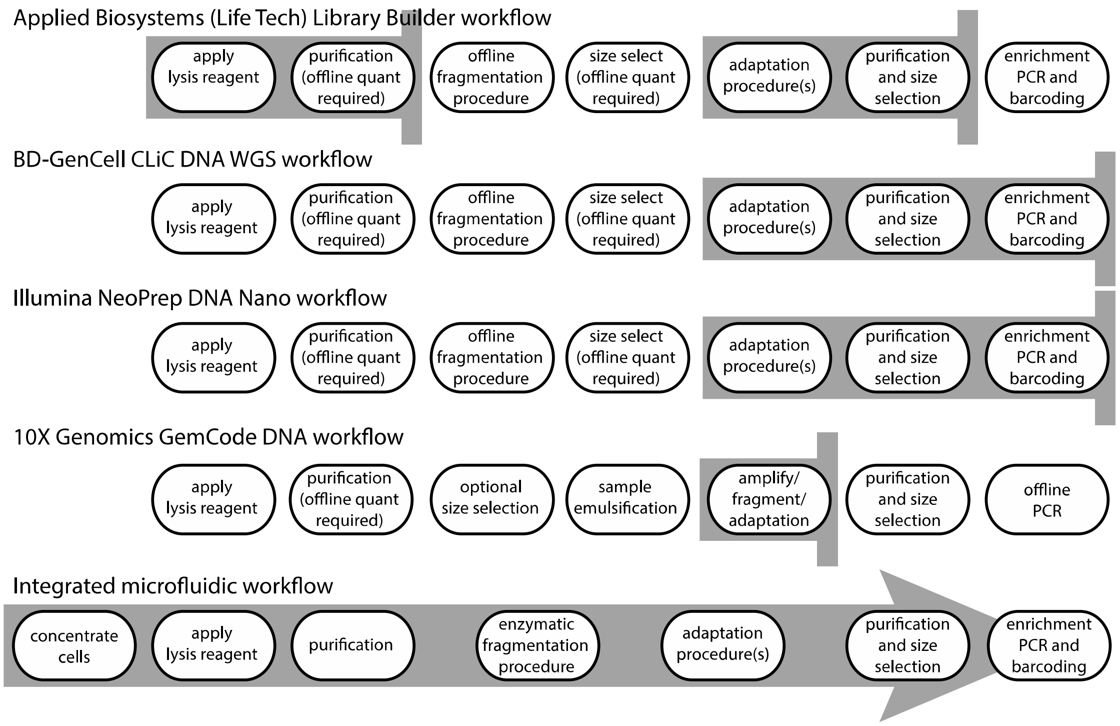

Supplementary Figure 2. Comparison of different WGS sample preparation technologies for Illumina NGS. No existing sample preparation systems have the ability to integrate the end-to-end WGS sample preparation. Our integrated microfluidic approach can perform the entire process end-to-end with full automation.

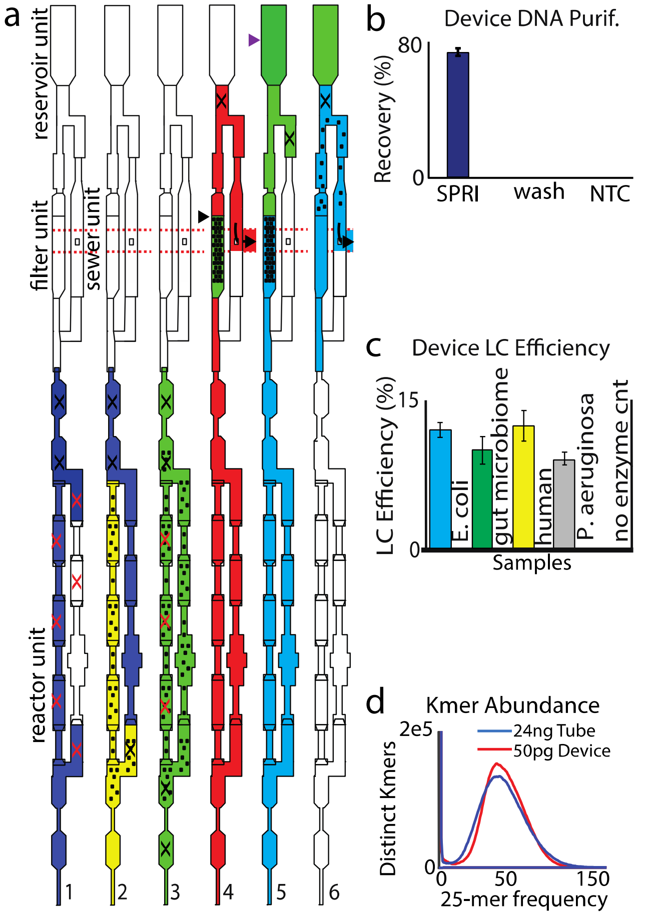

Supplementary Figure 3. Microfluidic workflow details, operation, and performance. a) Microarchitecture schematic and the workflows for the key operations; reagent mixing and bead capture. In the reactor unit, reagents are metered by the six valves (red X symbols) that can partition the ring into five 4 nL and one 16 nL compartment (blue and yellow colors represent different solutions and black dots represent SPRI beads in 1 & 2). These valves can also act collectively as a peristaltic pump to mix solutions (red X in 3 indicates pump; green color indicates homogenized mixture) around the reactor ring. We filter SPRI beads by flowing them through the filter valves (black arrow in 4) to create bead columns for washing (red and green in 4 are ethanol used to wash the beads and DNA molecules attached to the beads, respectively). Waste is evacuated through vias (black rectangle in the sewer unit) that connect the solution layer to the sewer layer (red dotted lines). Molecules are then eluted off the beads into the holding tank (purple arrow in 5; blue is solution used for elution). The used beads are flushed out into the sewer and the reactor and filter units are purged with air to prepare for the next reaction or purification operation. Black X designates valves in the closed state (details in methods). **b)** The on-device DNA purification efficiency (% of input recovered) from 50 pg gDNA is quantified by performing qPCR with adapter-conjugate primers on samples collected from the device after SPRI process before PCR amplification. NTC indicates no template control. Error bars indicate standard deviations (n = 4). **c)** The library construction efficiency for different types of DNA was calculated by quantifying the amount of (pre-amplified) tagmented product from the device by qPCR as described and dividing by the DNA input quantity. Error bars indicate standard deviations (n = 4). **d)** The number of 25-mer sequences at each abundance level is plotted to show the coverage distribution and frequency of error-containing low-abundance k-mers in conventional and low-input microfluidic libraries.

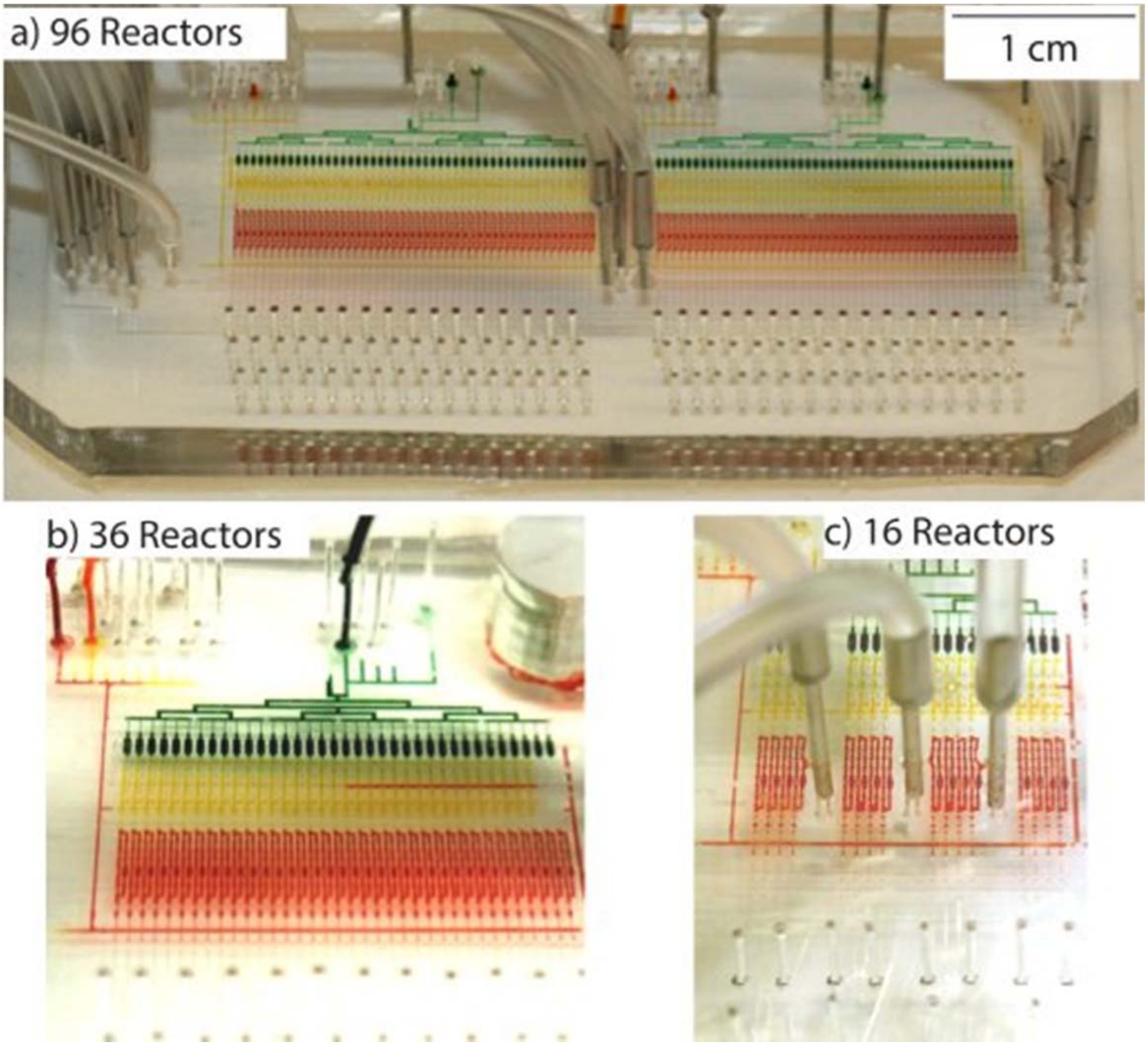

Supplementary Figure 4. Pictures of the microfluidic library construction devices. Three different prototype versions of the microfluidic library construction platform (96, 36, and 16 reactor and filter valve units) were used to produce the data for this study. All microarchitectural features are identical between the different capacity devices. Apparent feature distortion is due to optical refraction through the upper surface of the device.

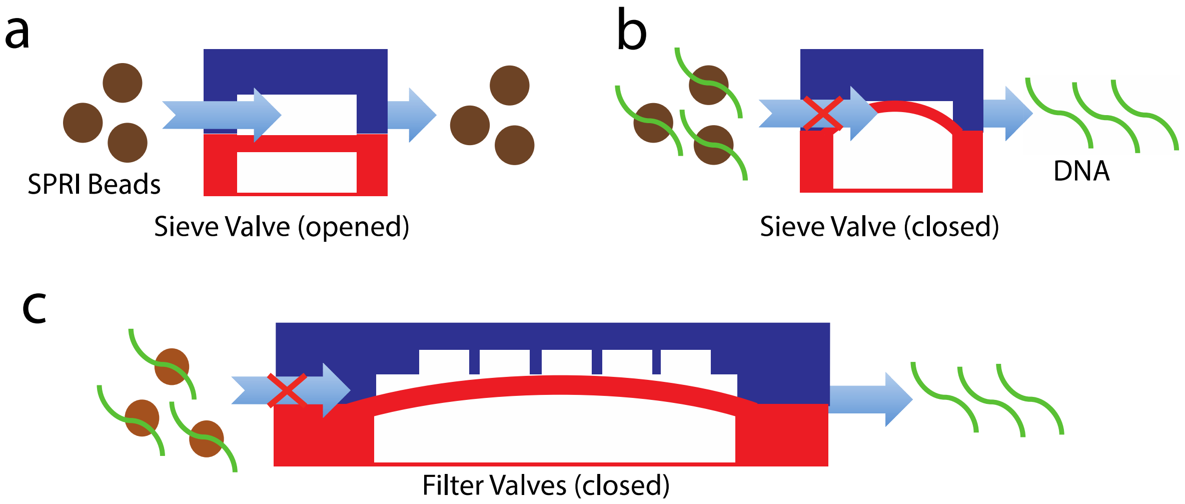

Supplementary Figure 5. Filter valves for solids handing and nucleic acid purification. Cross section of a sieve valve[1] in a) an open state and in b) a closed state. The blue arrows indicate the direction of fluid flow that is perpendicular to the cross section of the channels. In the closed state, the sieve valve blocks the beads that are larger than the pores created at the corners of the fluid channels (blue), while allowing passage of DNA fragments upon elution. There are only two pores per valve, which creates large flow resistance and is susceptible to clogging. c) The filter valves we introduce here have extra micro-channels that decrease resistance and enable device operations involving bead capture, washing, and elution with capability for handling significant quantities of solids.

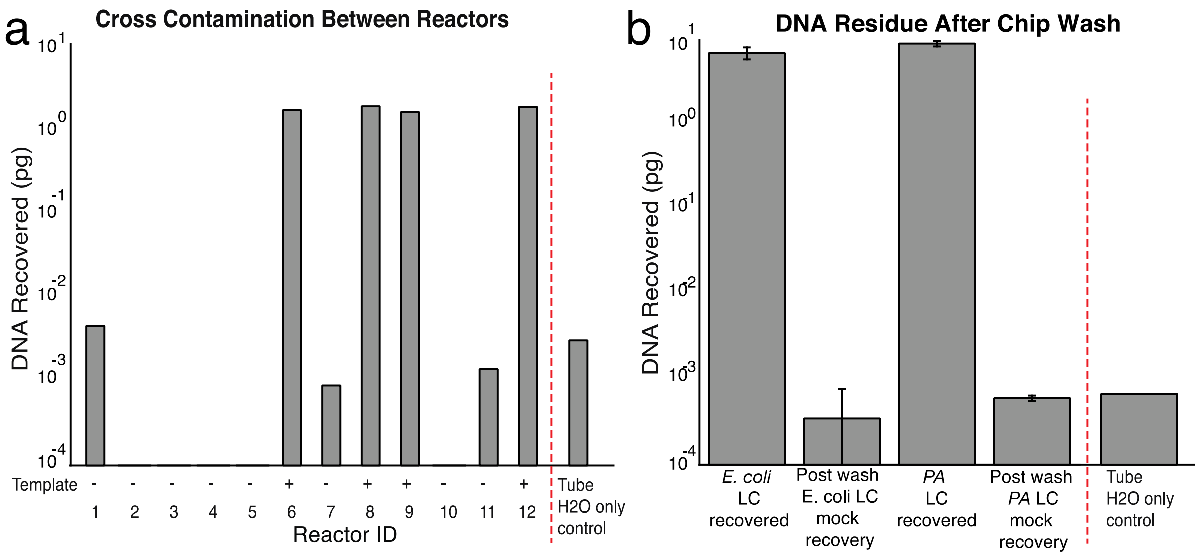

Supplementary Figure 6. Analysis of cross-contamination and carry-forward contamination on the microfluidic platform. a) Cross-contamination within a run was quantified by performing on-device SPRI purification of gDNA samples interleaved with no template controls, then quantifying the output by qPCR. The cross-contamination level between reactors was below the detectable limit for 5/8 no template controls and the remainder was measured to be ~10^{-3}, similar to the level observed in negative control (“tube H_2O only control”) qPCR reactions. **b)** We measured residual library fragments after a library construction run by flowing buffer through the device and performing qPCR. *PA* indicates *P. aeruginosa*. The reduction in DNA after the device wash was > 10,000 fold. The background level measured in this experiment was ~7 x 10^{-4} pg, while libraries made were in the pg range. Error bars indicate standard deviations (n = 4).

a Library fragment size distribution (from Bioanalyzer)

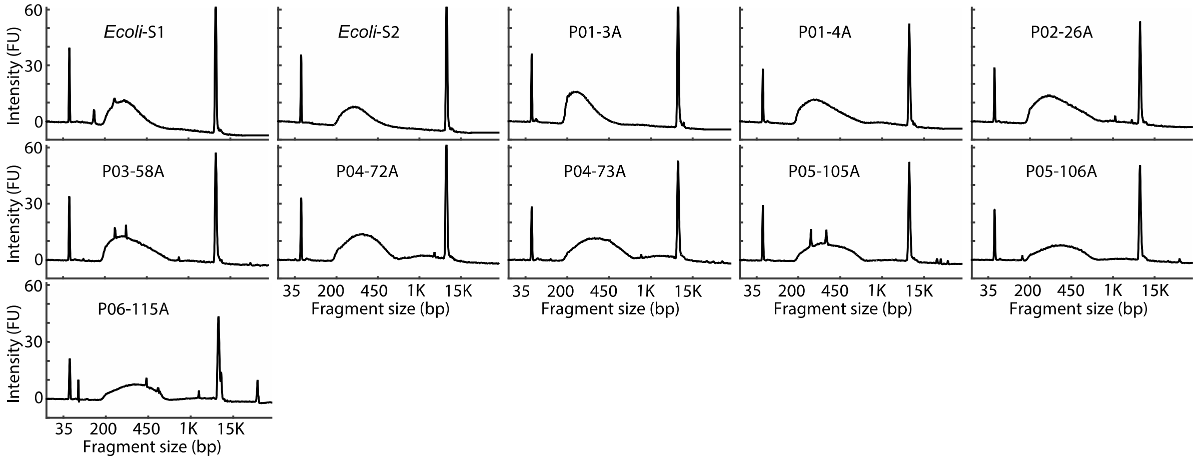

b Sequencing statistics

E. coli-S1 (ATCC 11303)

Total Reads	2,376,488
Duplicates	6.15%
Mapped	98.47%
Properly Paired	99.46%
Avg Cvg	53
Cvg SD	16.19
Cvg Coeff Var	0.304

P. aeruginosa-P01-4 (PA14)

Total Reads	2,337,181
Duplicates	3.38%
Mapped	89.48%
Properly Paired	99.06%
Avg Cvg	37
Cvg SD	14.42
Cvg Coeff Var	0.387

P. aeruginosa-P01-4 (*De novo* assembled P01-4)

Total Reads	2,337,399
Duplicates	2.46%
Mapped	98.86%
Properly Paired	98.95%
Avg Cvg	30
Cvg SD	11.29
Cvg Coeff Var	0.364

P. aeruginosa-P01-4 (*De novo* assembled P01-21)

Total Reads	2,337,413
Duplicates	2.20%
Mapped	98.68%
Properly Paired	98.93%
Avg Cvg	30
Cvg SD	11.62
Cvg Coeff Var	0.378

c Coverage distribution

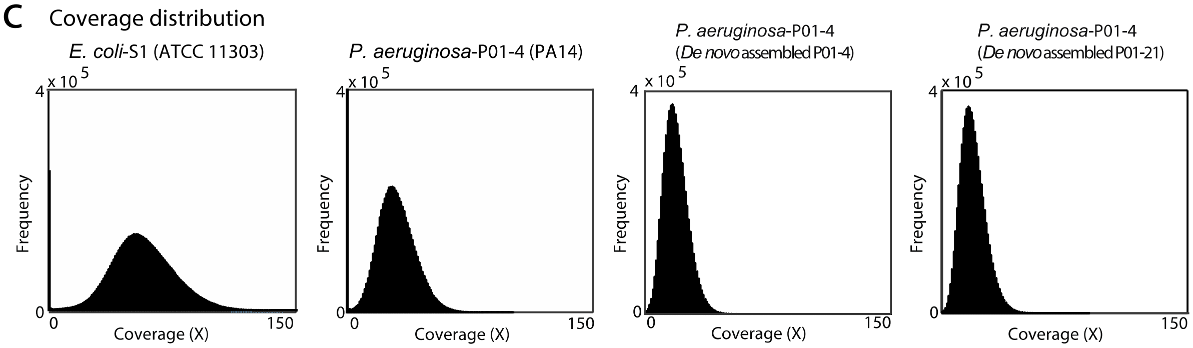

d Insert size statistics (from sequencing reads)

E. coli (ATCC 11303)

n = 3	mean	SD
mean insert size	155.40	3.84
SD insert size	66.28	1.58

P. aeruginosa (PA14)

n = 372	mean	SD
mean insert size	161.52	15.60
SD insert size	81.67	9.97

Supplementary Figure 7. Insert size and sequence coverage statistics. a) The library fragment size distribution for two *E. coli* samples and nine clinical *Pseudomonas* samples (Agilent Bioanalyzer). The notation “P01-4” indicates subject 1, sample 4. **b)** Sequencing statistics for one *E. coli* sample (S1) mapped to ATCC 11303 reference genome from Genbank and one of the *P. aeruginosa* samples (P01-4) mapped to three different reference genomes: PA14 from Genbank, *de novo* assembled P01-4, and *de novo* assembled P01-21. **c)** The coverage distribution plot of one of the *E. coli* samples (S1) and one of the *P. aeruginosa* samples (P01-4) mapped to three different reference genomes; PA14 from genbank, *de novo* assembled P01-4, and *de novo* assembled P01-21. **d)** The mean and the standard deviation of the read insert size of all *E. coli* and *P. aeruginosa* samples sequenced are reported.

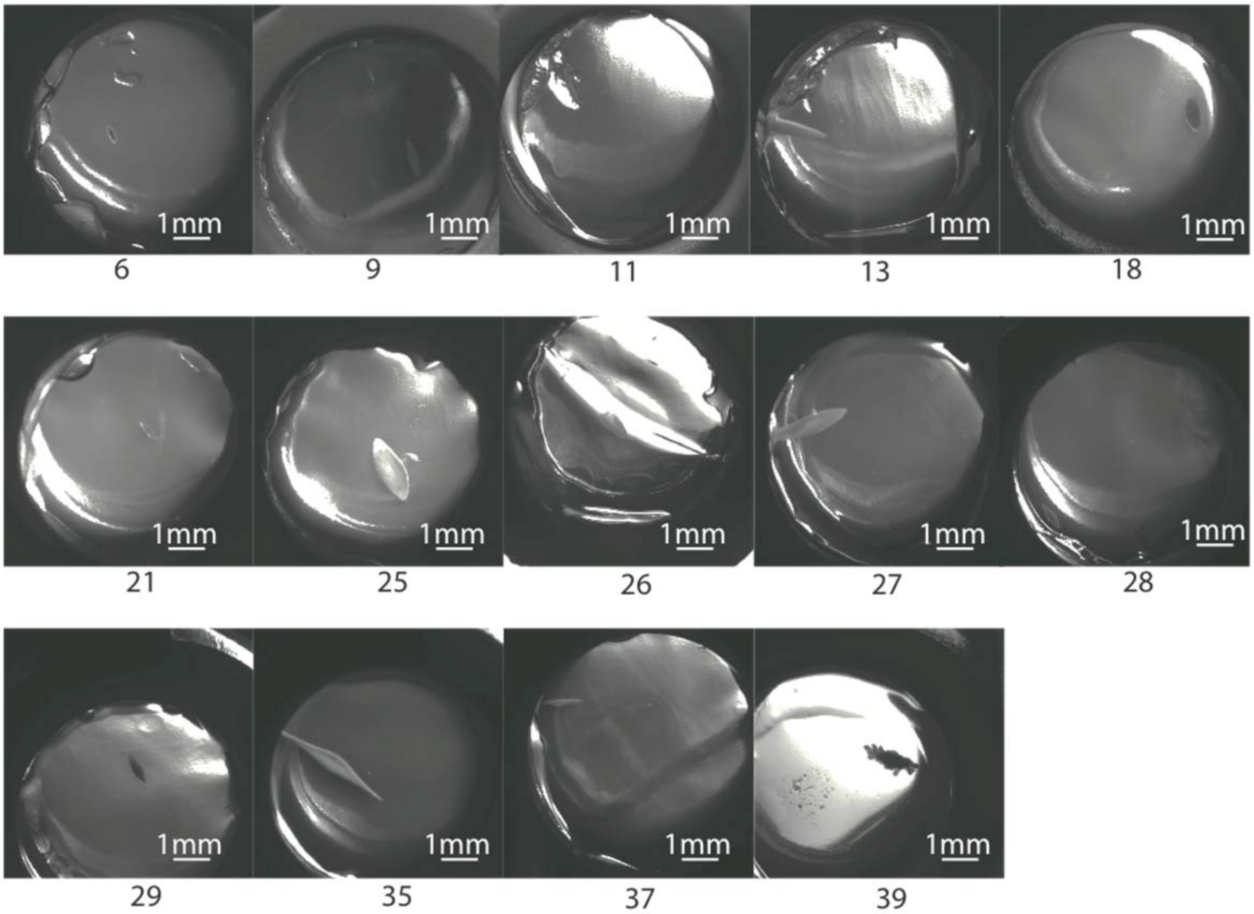

Supplementary Figure 8. Images of microcolonies cultured in the iChip. Image of soil bacteria microcolonies cultured using the iChip. Numbers below each image indicate sample index.

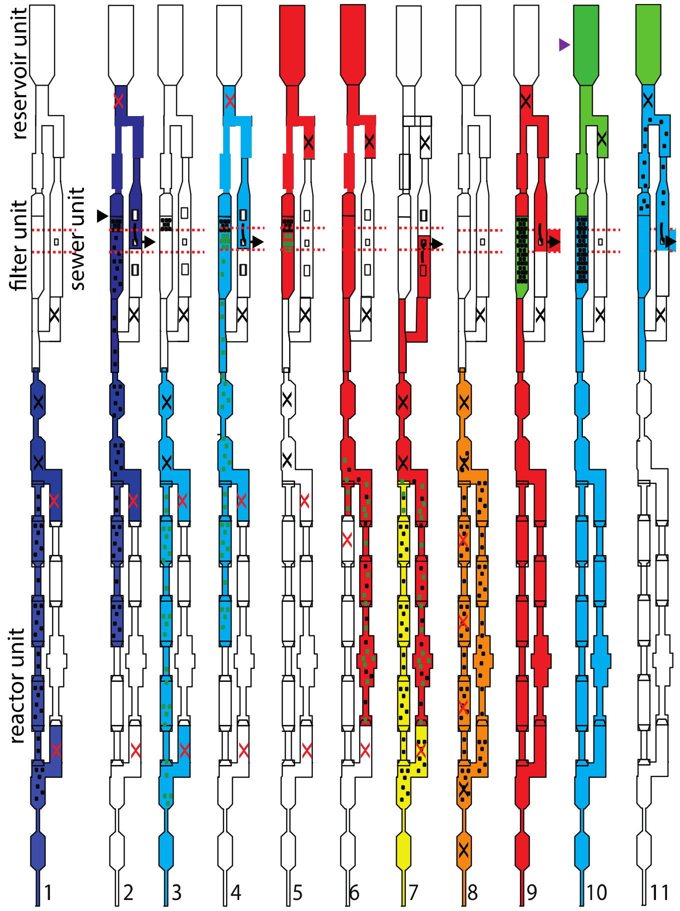

Supplementary Figure 9. Microfluidic cell lysis and DNA extraction workflow details for low-input cells. a) Microarchitecture schematic. The red and black “X” indicates valves at the closed state (details in methods). 1) & 2) A bead solution (blue background and black dots) is strained by the filter valve unit (black arrow in 2) to create a bead column, where the waste is evacuated through vias (black rectangle in the sewer unit) that connect the solution layer to the sewer layer (red dotted lines). 3 & 4) Through the bead column, a dilute cell solution (light blue background and green dots) is strained to concentrate bacteria cells in the filter unit. 5 & 6) The packed cells are pre-treated with 80C heat shock and loaded into the right side of the rotary reactors with DNase, hydrolyzing enzymes (proteinase K, mutanolysin, and lysozyme), and detergent lysis cocktail (red). 7 & 8) After incubating at 37C, 56C, and 80C for fragmentation, lysis, and heat treatment, SPRI beads and binding buffer (yellow background and black dots) are added into the reactor and mixed (orange color in 8 designates homogenized mixture) around the reactor ring. 9) We then filter SPRI beads by flowing them through the filter valves to create bead columns for washing (red and green in 9 represent ethanol used to wash the beads and DNA molecules attached to the beads, respectively). 10) Molecules are then eluted off the beads into the holding tank (purple arrow in 10; blue is solution used for elution). 11) The used beads are flushed out into the sewer and the reactor and filter units are purged with air to prepare for the next reaction or purification operation.

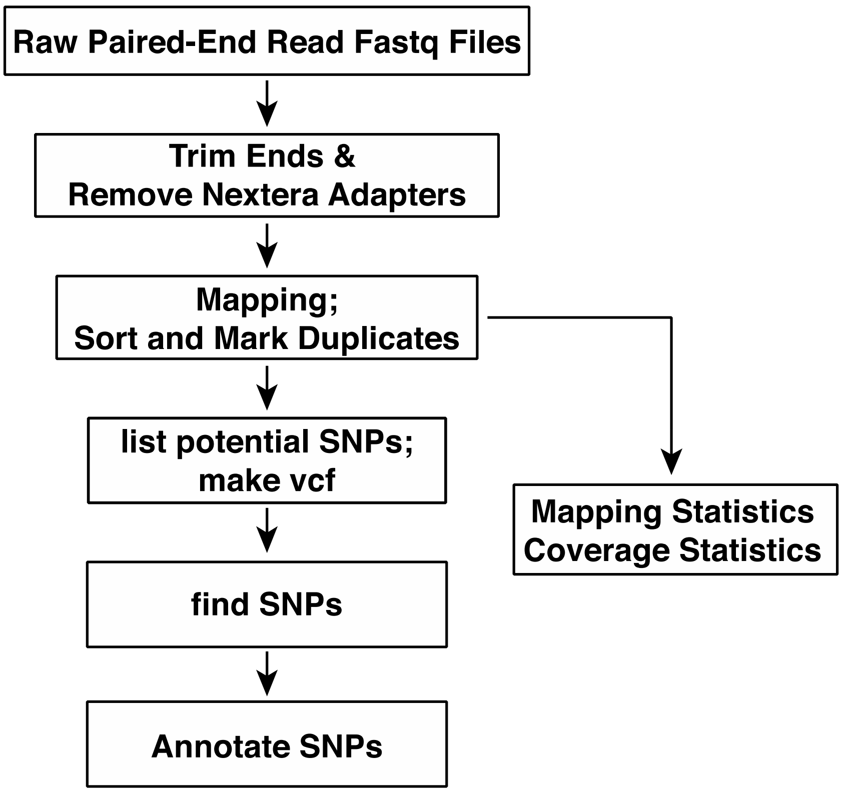

Supplementary Figure 10. Flowchart summarizing base calling procedure. Further details on the base calling procedure are included in methods and supplementary software files.

a

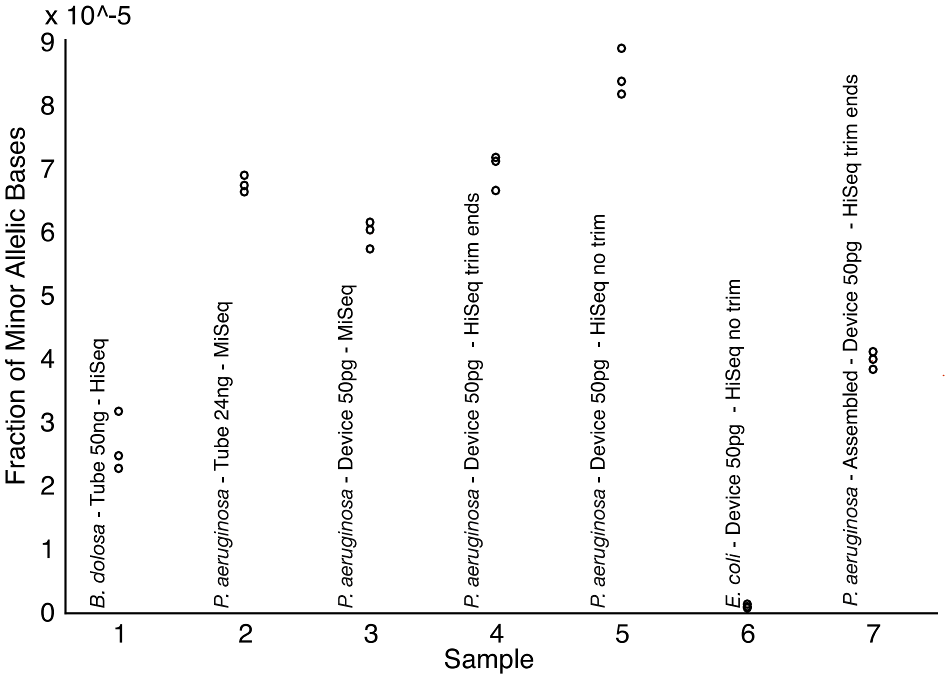

b

Plot no.; descr		Sample ID	Reference	Cov	Map %	GC %	LC	Seq	Trim ends	Pool
1	*B. dolosa*	P01-1,2,3	AU0158	15X	96.1	65	Tube 50 ng	Hiseq 1x50	no	N/A
2	*P. aeruginosa*	P01-1,2,3	PA14	19X	87.9	66	Tube 24 ng	Miseq 2x80	no	10
3	*P. aeruginosa*	P01-1,2,3	PA14	20X	89.3	66	Device 50 pg	Miseq 2x80	no	10
4	*P. aeruginosa*	P01-1,2,3	PA14	66X	90.3	66	Device 50 pg	Hiseq 2x125	yes	384
5	*P. aeruginosa*	P01-1,2,3	PA14	89X	90.3	66	Device 50 pg	Hiseq 2x125	no	384
6	*E. coli*	S1,2,3	ATCC11303	45X	98.4	50	Device 50 pg	Hiseq 2x125	no	384
7	*P. aeruginosa*	P01-1,2,3	Assembled	56X	98.3	66	Device 50 pg	Hiseq 2x125	yes	384

Supplementary Figure 11. Fraction of minor allelic bases in raw read data. a) Dot plot of the fraction of minor allelic bases that likely accrue from sequencing error. No minor allelic bases are expected since the samples originated from single colonies. Three samples from each category were selected for analysis. **b)** Table that lists the sample descriptions and variables tested for contribution to the error. *B. dolosa* data is from T. Lierberman *et. al*[2]. The notation “P01-1” indicates subject 1, sample 1.

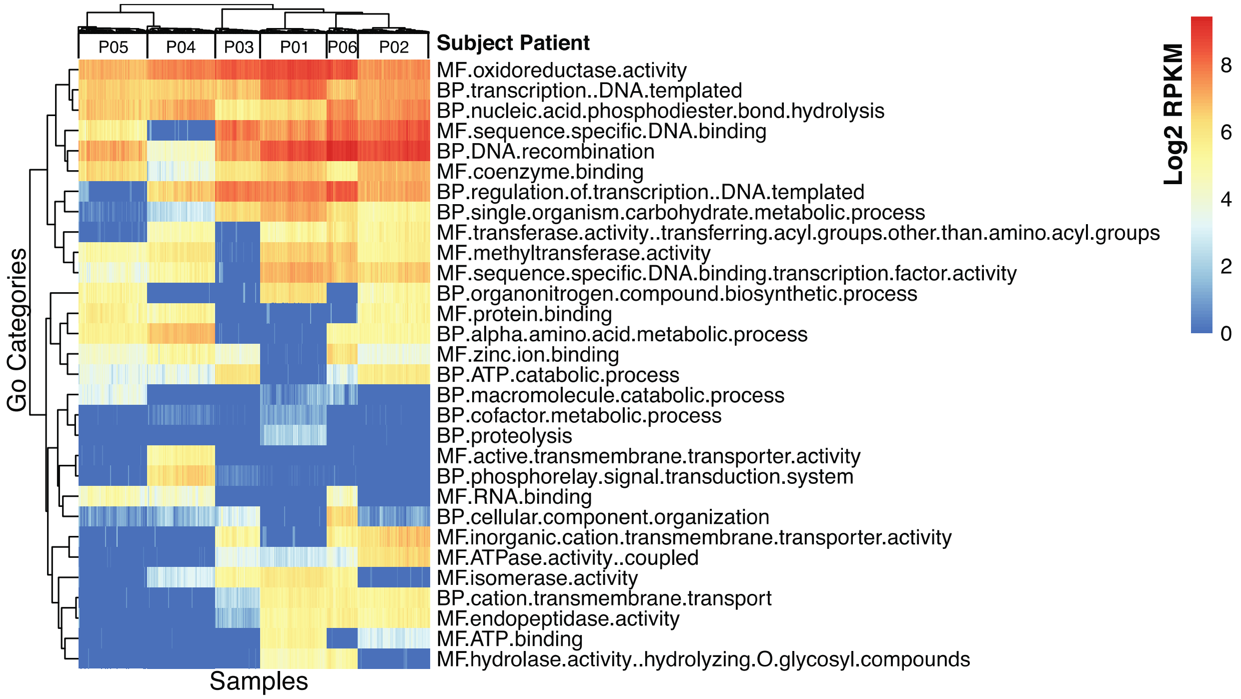

Supplementary Figure 12. Variable genome analysis. Gene content analysis was performed on the ~1500 genes that varied the most between subjects. This heat map reports the number of reads that map to each gene category (rows) in the *P. aeruginosa* pangenome. Gene categories are labeled by molecular function (MF) and biological processes (BP). This analysis was generated using HUMAnN2 and the genes were clustered according to GO categories (methods). After unsupervised clustering of the results, each of the 124 *P. aeruginosa* samples was grouped according to subject of origin. RPKM stands for reads per kilobase DNA per million mapped reads.

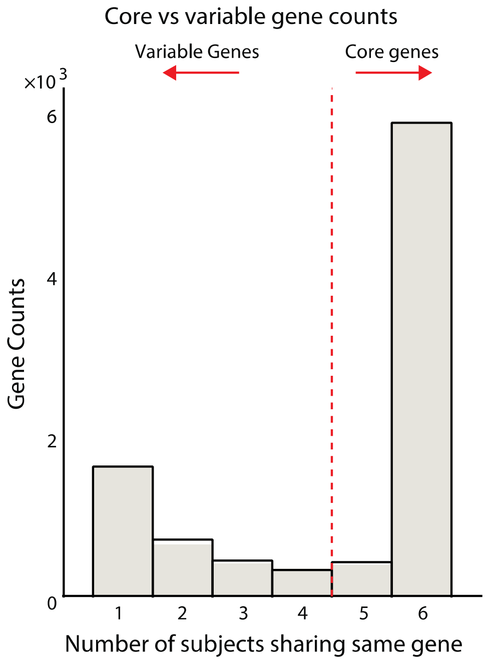

Supplementary Figure 13. Gene set enrichment analysis of the core and variable genome of *Pseudomonas* isolates between patient subjects. a) Histogram of genes shared across subject strains, identified by pangenome analysis. There were 6000 core genes (shared by ≥ 5/6 subjects) and 3000 variable genes (shared by ≤ 4/6 subjects). The red dotted line indicates the breakpoint between core and variable genes.

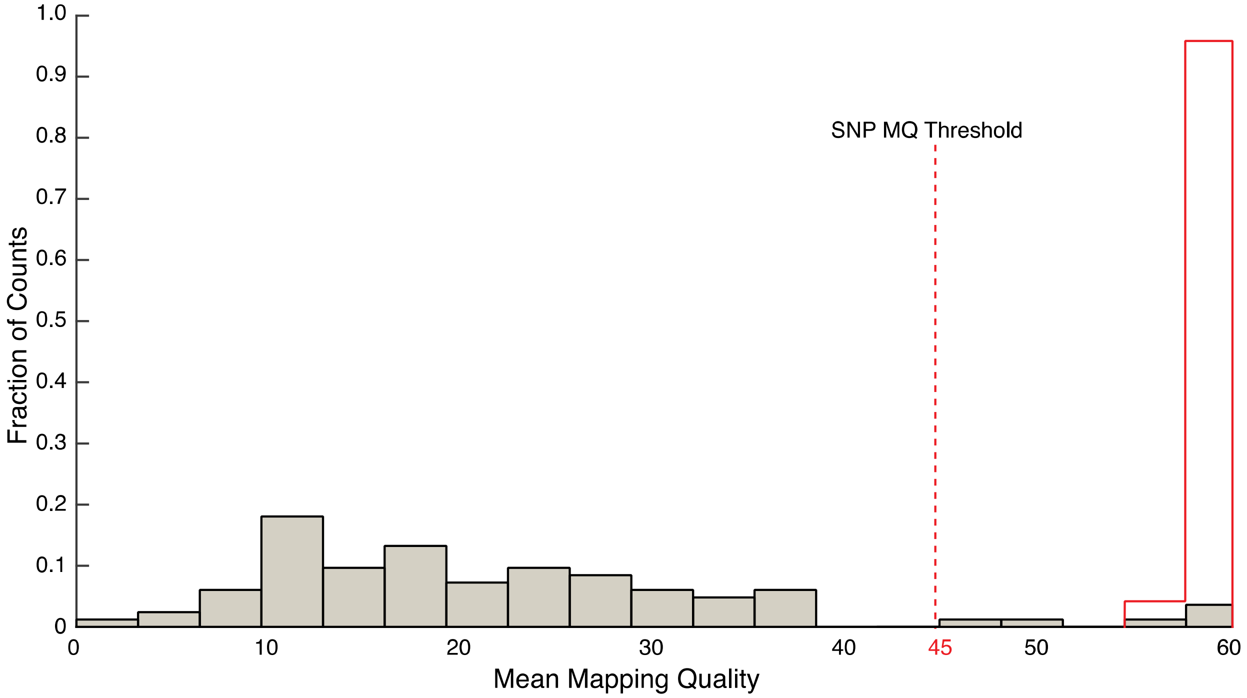

Supplementary Figure 14. Mapping quality (MQ) value analysis. The distribution of mean MQ values (grey filled bars) for sites with read depth > 6 and AF > 0.82 where projected base calls differ among isolates from the same clinical *Pseudomonas aeruginosa* study subject, compared to the distribution of mean MQ values at SNP sites tested to confer drug resistance (red outline bars). A threshold at MQ = 45 (red dotted line) is used to eliminate sites with low MQ values before calling SNPs.

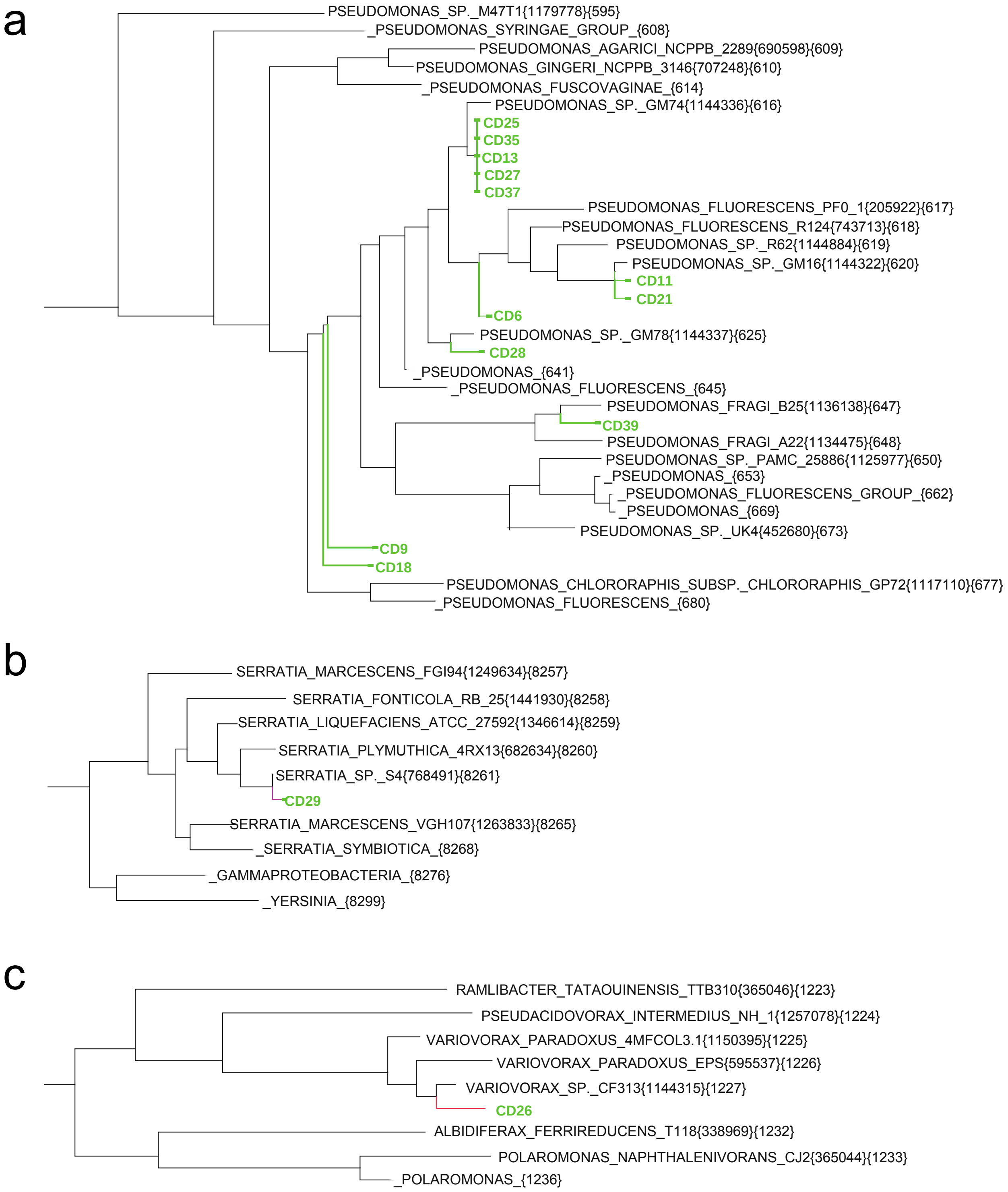

Supplementary Figure 15. Soil micro-colony phylotyping. The soil micro-colonies that were processed in the microfluidic device and WGS sequenced were *de novo* assembled using SPAdes[2], then phylotyped at the stain level using Phylosift[3], a software tool for multi-locus sequence typing analysis.

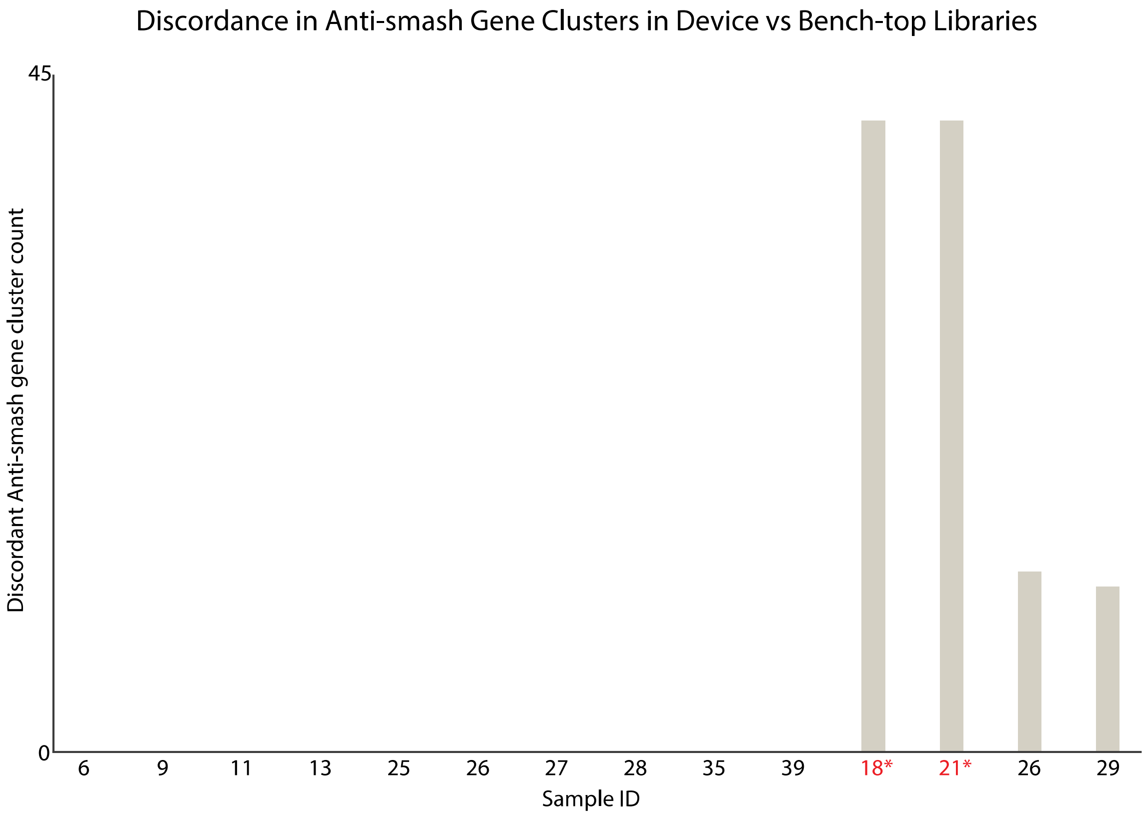

Supplementary Figure 16. Micro-colony secondary metabolite gene cluster prediction difference between device and bench-top libraries. Metabolic cluster analysis using the antiSMASH pipeline yielded diverging results for 4/14 micro-colonies. These samples (18, 21, 26, and 29) showed much higher library complexity in the device libraries than the bench-top libraries (893, 464, 577, and 141 fold coverage in the device libraries versus 7, 13, 316, and 24 fold coverage in the bench-top libraries). The sequencing data for two of the bench-top libraries (samples 18 and 21; red asterisks) did not have sufficient complexity for *de novo* assembly or anti-smash analysis, although the device libraries for these two samples produced sequence data indicating excellent complexity (Figure 1f; Supplementary Data 9).

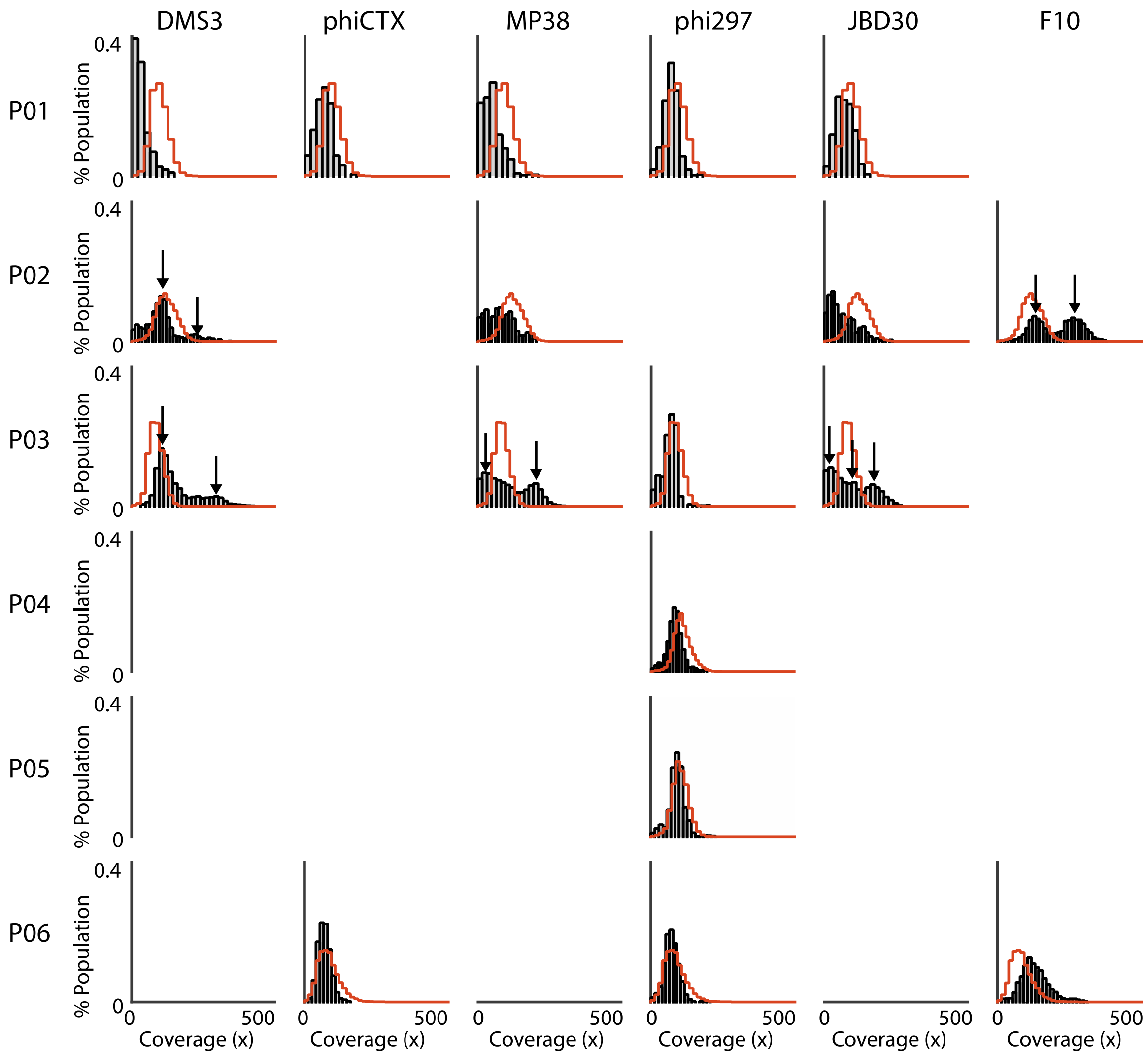

Supplementary Figure 17. Phage coverage histogram. The genome coverage histograms from one of the isolates from each subject. The histograms are reads mapped to six different *Pseudomonas* phage genomes (DMS3, phiCTX, MP38, phi297, JBD30, and F10) that were identified to be present in our dataset by Metaphan 2.0. The blank entries represent cases where less than 0.05% coverage for the specific phage genome was obtained. The red line in each plot represents the *P. aeruginosa* reference genome (PA14) coverage distribution for the isolate under analysis. While most phage coverage histograms had nearly identical coverage and distribution to that of the coverage histogram of the PA14 genome, P02 DMS3, P02 F10, P03 DMS3, P03 MP38, and P03 JBD30 had multiple subpopulations with tight integer-varied multi-modal coverage distributions (black arrows).

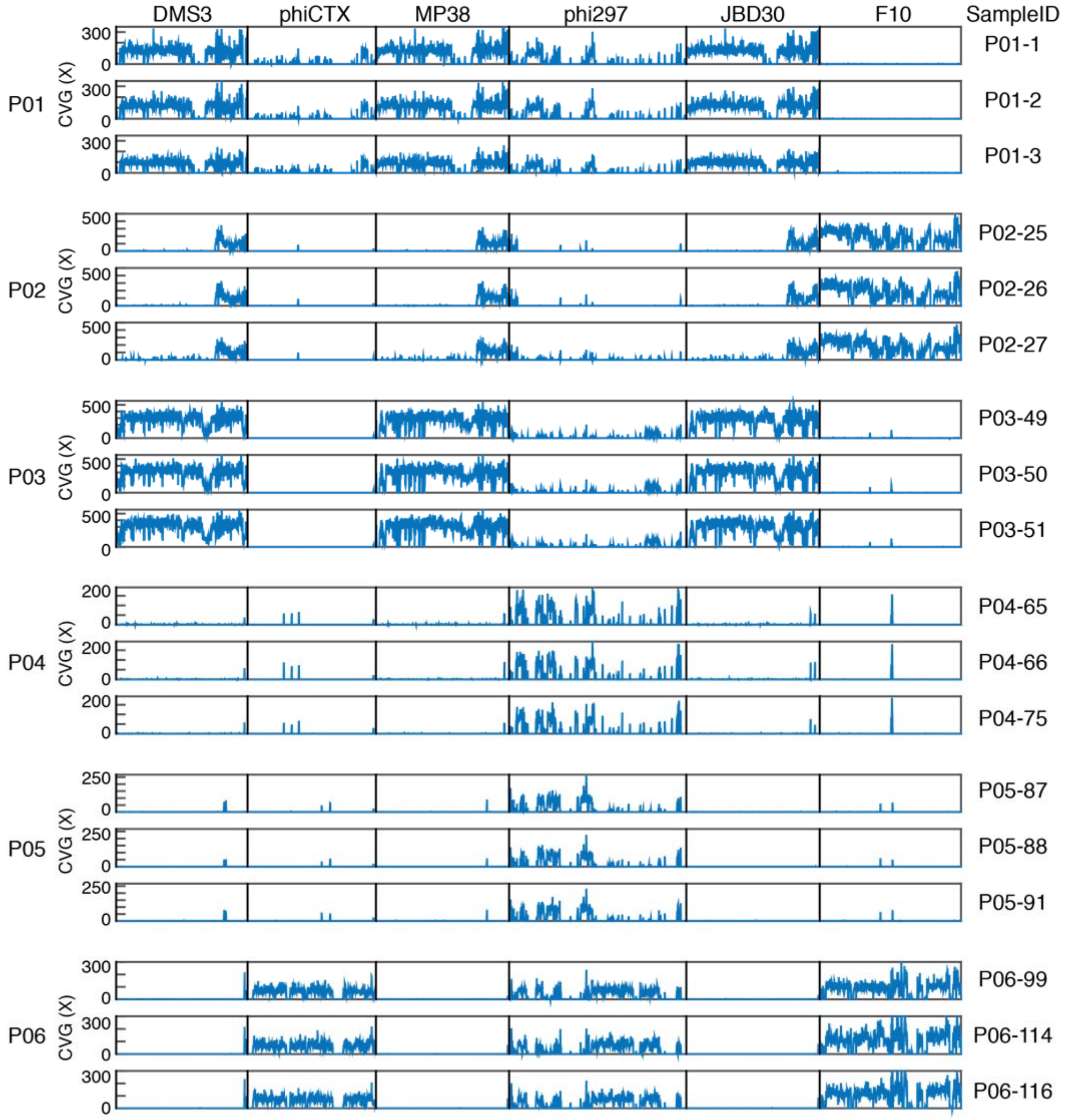

Supplementary Figure 18. Phage genome coverage plot. The phage genome coverage plot from three sample isolates from each subject. The graphs plot the sequencing coverage along the positions of the six different *Pseudomonas* phage genomes (DMS3, phiCTX, MP38, phi297, JBD30, and F10). Distinct coverage features specific to each subject, including large deletions, are evident.

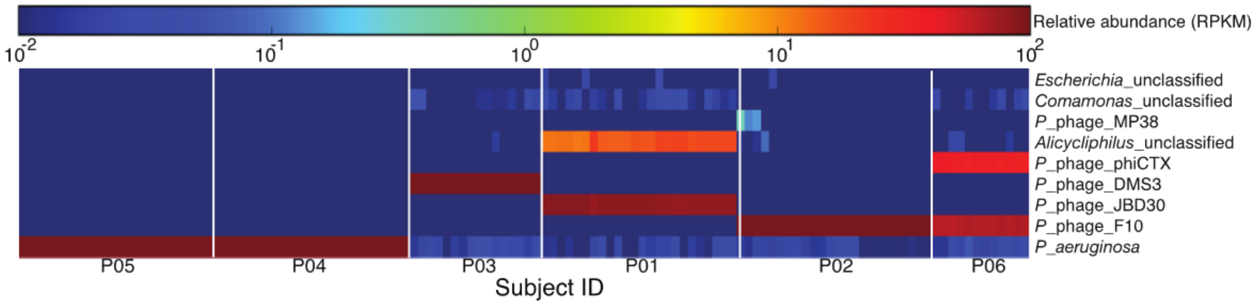

Supplementary Figure 19. Novel *Pseudomonas aeruginosa* variable genome content in the clinical isolate samples. Analysis of reads that did not map to the *Pseudomonas aeruginosa PA14* reference genome (Metaphlan 2.0) identifies *Pseudomonas* phage and sequences closely related to other bacteria that cluster by subject. The columns are isolates that are grouped (unsupervised) by the patient subject.

Supplementary Note 1. Solid-phase reversible immobilization (SPRI). SPRI[4] is a DNA precipitation-based cleanup method that has become ubiquitous in genomics sample preparation. SPRI is robust across various types of crude samples, allows for DNA fragment size selection, and can be automated on liquid-handling robots. SPRI purification requires a capability to retain beads while exchanging solutions, and is often implemented using magnetic particles and externally applied magnetic fields.

Supplementary Note 2. Analysis of cross-contamination and carry-over contamination. We verified that sample cross-contamination across reactors on the device remains undetectable within our limit of quantification by carrying out qPCR on products from microfluidic negative-control reactions (Supplementary Figure 6a). To test whether carry-over contamination would occur if the device were re-used, we quantified residual library fragments recovered from a used device, finding that the residual DNA level was reduced more than 10^4 fold by rinsing the device between runs (Supplementary Figure 6b). The low amount of residual DNA indicates that carry-over contamination attributable to the device would be comparable to typical carry-over levels on NGS platforms themselves (~10^{-4}).

Supplementary Note 3. Library complexity estimation with sequencing data. The complexity was estimated using a shotgun sampling model[5]. The calculations for one 1000 cell *E. coli* sample library and one clinical *Pseudomonas* isolate sample library are given here as examples. We quantified *E. coli* cells on a hemocytometer and obtained an estimate of the genomic content in the cell sample consistent with approximately 5 fg gDNA per cell (quantified by qPCR) in our late log phase culture. The *P. aeruginosa* DNA was quantified by the Qubit fluorescence DNA quantification kit.

Picard tools (http://broadinstitute.github.io/picard/) uses the following equation[5] to estimate the library complexity:

$$d = 1 - \frac{N}{m}\left(1 - e^{-\frac{m}{N}}\right)$$

where *d*, *N*, and *m* are duplication rate, number of unique molecules in the library, and total reads sequenced, respectively.

Unique molecules = # of unique adapted molecules present in our library before amplification

Library content (in base pairs) = (unique molecules) x (mean insert size)

Library complexity (in genome equivalents/fold-coverage/"x") = $\frac{\text{Library content (bp)}}{\text{genome size (bp)}}$

Library conversion efficiency = $\frac{\text{Library complexity}}{\text{gDNA Input}}$

* GE = genome equivalents

Organism	*E. Coli*	*P. aeruginosa*
Reference genome	BL21-DE3	PA14
Sample ID	1000 *E. coli* cells	P02-26B
Genome size (bp)	4.61E+06	6.60E+06
Library construction	Device 5 pg	Device 50 pg
DNA input (pg/GE)	5 pg (1000 GE)	50 pg (6910 GE)
Mean insert size (bp)	126.3	156.9
Total reads	16,993,424	2,814,581
Duplication (%)	60.61%	2.81%
Unique molecules	7.46E+06	4.91E+07
Library complexity (GE)	102.19	583.62
**LC efficiency (%)**	**10.22**	**8.45**

The theoretical maximum efficiency for Nextera chemistry is 50% based on random combinations of conventional A/B sequence adapters, and we think 30 – 35% efficiency is practically achievable in our system with modifications to the operating protocol. Increased efficiency would allow more stringent size selection of the library, greater library complexity, and/or lower input quantity.

Supplementary Note 4. Estimation of soil micro-colony cell quantities loaded and processed in the device. The number of cells in each colony was not directly measured because the entire sample collected was needed for sequence library preparation. The micro-colonies (Supplementary Figure 8) were hundreds of microns to millimeters across. Given the rod like shape of *Pseudomonas* cells with dimensions of 0.5 ~ 1 x 1.5 ~ 5 μm, we estimated that the micro-colonies contain a total of 10^4 -10^6 cells depending on the three-dimensional shape of the micro-colonies. A fraction of each micro-colony was sampled by toothpick. We estimate that 10% to 50% of the cells in each micro-colony were sampled using our procedure, which was optimized to minimize contamination of the cells with the agar substrate from the iChip.

Based on the library complexity estimates from the sequencing data (Figure 1f) and the fragment sizes of the libraries, we estimate ~10^4 lysed cells as input for the on-device tagmentation reaction. The amounts of cells accumulated on the filter valves during the cell concentration process were also visually comparable to ~10^4 *E. coli* cells from previous experiments (data not shown). Overall, this was consistent with a significant fraction of the colony being delivered to the device and efficiently lysed.

Supplementary Note 5. Optimized low-input DNA extraction method. To determine the contamination levels and sequence library quality of our device libraries we carried out a matched comparison of micro-colony sequence libraries prepared on the bench-top by an optimized low-input procedure closely related to the microfluidic method (using the same reagents in the same laboratory environment by the same operator). We used the custom optimized sample preparation method because commercial kits that use column-formatted DNA capture/purification steps are known to provide poor yields from the extremely low inputs[6] (<10 pg) used in this study (bead-beating DNA extraction at low-input produced output indistinguishable from negative control, data not shown). The enzyme and detergent selection,

temperature, and buffer conditions were optimized for lysis efficiency and compatibility with downstream steps. Our protocol performed equally or better than commercial kits (including bead-beating kits) for higher-input bacterial samples (data not shown).

Supplementary Note 6. Consensus base calling accuracy and types of errors, and replicate sequencing to perform error correction. Typical error rates in short-read NGS are 1e-3 for raw bases and 1e-5 for standard consensus sequencing[7]. Consensus base calling errors in NGS can be attributed to processes occurring at four stages in the WGS sequencing process workflow: 1) sample handling, 2) library construction, 3) sequencing, and 4) analysis[8]. Sample handling factors affecting accuracy include sample labeling mix-ups and sample degradation[9]. Library construction errors can accrue from mutations and chimera formation in amplification steps. Sequencing errors such as incorrect raw base calls have platform-specific frequencies and correlations. Analysis errors include improper read mapping and local alignment errors. The depth and variance of read coverage are also important considerations in consensus base calling. Ideally, library construction produces fully random fragments without bias, and the sequencing platform has equal sensitivity and accuracy for all fragments and all bases. If these fragments can be mapped accurately, ideally randomized coverage of the template will be achieved without mismapped reads. We prepared three libraries from each *P. aeruginosa* colony in our study and sequenced these replicates independently to enable the detection of sample preparation errors and to evaluate the ability of replicate sequencing to correct possible library construction and sequencing errors (Figures 2b-d).

Supplementary Note 7. Pilot study control samples. Controls in the pool of 384 samples for the clinical *P. aeruginosa* study included three device wash samples (that report residual library molecules remaining in the device after the LC process) and three *E. coli* samples (Figure 1c). The chip wash samples produced few reads, while the *E. coli* reads mapped poorly to the PA14 genome but mapped at a rate > 99% to the *E. coli* ATCC 11303 reference genome (Figure 1c, last three samples & Supplementary Figure 7).

Supplementary Note 8. Pooled sequencing run of 384 clinical *Pseudomonas* isolate samples. We used custom dual-indexing Nextera primers designed by the Broad Institute Genomics Platform to barcode our 384 samples. After barcoding each of our samples during enrichment PCR, we pooled all 384 samples to a single mixture by normalizing individual samples based on their concentrations. Pooling high numbers of samples in a single sequencing flow cell raises the possibility of read mis-assignment that results in consensus errors. We evaluated the possibility of mis-assignment by evaluating the fraction of minor allele bases occurring in the 384-sample-pooled *P. aeruginosa* HiSeq runs versus a 10-sample-pooled *P. aeruginosa* sequencing run where barcode mis-assignment is far less likely (Supplementary Figure 11). Each *P. aeruginosa* sample was from a single colony, thus should have no minor allelic bases in the absence of technical errors such as barcode crosstalk. Two pieces of evidence allow us to exclude barcode swaps as a significant contributor to the observed minor allele frequencies in our *P. aeruginosa* samples: 1) we observe a similar incidence of minor allele bases in the large and small pool and 2) alignment errors appear to contribute most of the observed minor allele bases, as the ATCC 11303 *E. coli* sample in the 384 sample pool (for which an ideally-matched reference was available) shows a far lower minor allele frequency than the clinical *P. aeruginosa* samples (where accurate read mapping is more challenging).

Supplementary Note 9. Clinical *Pseudomonas aeruginosa* isolate diversity (SNPs). The continuous distribution of allelic fraction (AF) values is a general challenge in variant calling. To

examine sources of broadening in our *P. aeruginosa* AF distributions, we looked at the fraction of minor allelic bases sequenced at each position of the genome as a function of GC content, sequencing quality, mapping rate, and DNA input (50 pg in the device vs 24 ng in conventional libraries) (Supplementary Figure 11). The results show that AF broadening is associated with the sequencing instruments/kits, GC content, and mapping rate, but no difference was found between low-input microfluidic libraries and conventionally prepared bench top libraries.

Sequencing error is known to increase as a function of read length. By trimming the ends of the *P. aeruginosa* HiSeq reads at the length where the sequencing base quality, reported by the sequencer, dropped exponentially reduced the minor allelic fractions by 20%. We also suspected mapping accuracy as another source of error. *P. aeruginosa* strains typically contain ~10% variable genome content[10], which poses a challenge in selecting the correct reference genome to align to in studies that involve multiple strains. In fact a significant reduction (2 fold) of minor allelic base fraction was achieved when we mapped the *P. aeruginosa* reads to *de novo* assembled custom *P. aeruginosa* reference sequences, which resulted in a mapping rate higher than 98%. Higher quality *de novo* assembled references for each strain (Supplementary Data 13) and longer sequencing reads would enable the use of less stringent mapping quality (MQ) cutoffs.

While we observed some intra-subject SNPs that have extreme AF values (values near 0 or 1; in alignments to the common PA14 reference genome), other SNPs have intermediate AF values (Supplementary Data 5; Supplementary 14) likely attributable to variations in strain-specific mapping/alignment quality across different loci. There were two intra-patient SNP sites where variant calls were initially made for a minority of samples due to low AF values. Upon manual inspection of alignments at these loci, it was clear that the alignments had obvious problems including abrupt coverage jumps, binary genotype mixtures across reads, and much higher fractions of partially mapped reads and reads with unrealistic calculated insert sizes. These problematic loci initially appeared in our analysis because one isolate from subject P04 and one isolate from subject P06 exhibited such a locus that (barely) passed the read depth, average mapping quality, and AF thresholds we set. These regions were masked out based on our interpretation that such loci in the reference are not present in the subject P04 and subject P06 strains and that spurious alignments led to these variant calls. Sanger validation showed no evidence that these loci existed in the samples (data not shown).

Since only 90% of our data aligned to the PA14 reference genome, we checked for additional intra-subject SNPs in the remaining 10% of the reads by *de novo* assembling reference genomes with our data from each subject using the SPAdes assembler[2] (Supplementary Data 13). However, we found no additional SNPs in these remaining reads (data not shown).

Supplementary Note 10. *Pseudomonas aeruginosa* isolate antibiotic susceptibility. To determine how our genomic findings corresponded to the isolate phenotypes, we determined the susceptibility of isolates to three antibiotics: imipenem, ciprofloxacin, and ceftazidime. We randomly selected a subset of isolates from each subject and measured their antibiotic resistance using the Kirby-Bauer disc diffusion susceptibility test[11]. As with the genomic measurements, this test revealed substantial inter-subject phenotypic diversity and negligible intra-subject diversity (Figure 3a). All six subjects' isolates were susceptible to ceftazidime, but subjects P01 and P04 were resistant to imipenem and subjects P01, P03, and P06 were resistant to ciprofloxacin. 100% of the phenotypically observed variability in resistance was explained by the genomic variants detected in our analysis of low-input sequence libraries produced in high throughput and sequenced to 50x.

Supplementary Note 11. *Pseudomonas aeruginosa* isolate gene content and gene set enrichment analysis. We aimed to identify variability in both gene content and sequence across isolates within and between subjects. *P. aeruginosa* isolates can be highly variable, with ~10% of gene content differing between strains[10]. The *P. aeruginosa* pangenome, has been cataloged as a curated reference of the 44,000 genes identified to date[12]. Aligning reads from each isolate to the pangenome showed that isolates from a given subject had identical gene content while extensive differences existed between isolates from different subjects (Supplementary Figure 12). Genes commonly found in five or six of the six subject strains numbered about 6000 (the core genome), while genes missing in two or more subject strains totaled about 3000 (the variable genome) (Supplementary Figure 13). The core genome was enriched for translation-related genes, according to Gene Ontology (GO)[13] categories and UniRef50[14] based on biological process and molecular function annotations (Supplementary Data 6). In the variable genome, we found enrichment for genes with functions related to DNA transposition, recombination, restriction-modification, and transition metal nanoparticles/metal ion response genes tied to mercury resistance (Supplementary Datas 6 & 7).

Supplementary Note 12. *Pseudomonas aeruginosa* isolate diversity in acute versus chromic infection. We found our *P. aeruginosa* isolates between subjects to be diverse in gene content and genome sequence across subjects even though all samples were collected from the same hospital within a period of a few weeks, yet the isolates from each subject were clonal. In contrast to our results, diverse *P. aeruginosa* populations have been reported to arise within individual cystic fibrosis (CF) patients chronically infected with *Pseudomonas aeruginosa*[10,15–20]. The CF lung is a permissive and spatially structured environment that is commonly colonized with *P. aeruginosa* over a period of many years in a way that is nearly impossible to eradicate with antibiotic therapy, enabling *P. aeruginosa* to evolve and diversify[21]. Both longitudinal[18,22] and latitudinal[17] CF studies report substantial intra-patient variability and even greater differences among isolates from different patients.

The presumably acute infections assayed in our study demonstrated almost perfect homogeneity across samples collected from each individual subject. The ability to sequence multiple isolates from a patient cheaply and easily may help to track the spread of infections using molecular epidemiology approaches and also to identify those patients with minority isolates that express antibiotic resistance or virulence traits that would not be apparent in standard analyses.

Supplementary Note 13. *Pseudomonas aeruginosa* isolate variable genome novel content analysis. The adaptability of *Pseudomonas* to diverse environmental conditions is attributed to its plastic accessory genome[10,23]. It has been reported that *P. aeruginosa* gains and loses genes by exchanging genetic material with other bacteria as well as phages[23], and we observed strong enrichment for genetic mobility elements in the variable genome of our isolates (Supplementary Datas 6, 7, & 12; Supplementary Figure 13). We analyzed reads that did not map to the PA14 reference genome and predicted the taxonomic origin of these sequences using Metaphlan2[24] (Supplementary Figure 19). A significant fraction of these undetermined reads mapped to *Pseudomonas* phage markers with the exception of the subject 4 and 5 strains, whose reads mostly mapped to genes from other *P. aeruginosa* strains.

In *de novo* assemblies of the clinical strains (13), we found individual contigs in each subject that contain strong sequence homology hits to known *P. aeruginosa* genes and *Pseudomonas* phages or other bacteria such as *Alicycliphilus denitrificans BC* using BlastN (Supplementary Data 12). We also found that the coverage depth and distribution of the *Pseudomonas* phage reference sequences were nearly identical to that of the PA14 reference (Supplementary Figure

17). These observations led us to conclude that the phage sequences represent prophage integrated into the *P. aeruginosa* genome. It also caught our attention that there were some phage genomes that had tight integer-fold higher coverage, which could be explained by multiple transposition events (Supplementary Figure 17). When mapping to the full phage genomes (Supplementary Figure 18), we found coverage patterns, which were specific to individual subjects and reproducible across isolates from each subject.

Analyzing many individual isolates enabled us to resolve the distribution of phage sequences in each isolate. Without a method for high-throughput sequence sample preparation, researchers would likely analyze intra-subject *P. aeruginosa* diversity by pooling the isolates, which would make the distribution of phage sequence across individual isolates ambiguous.

Supplementary Note 14. Outlook for microfluidic sample preparation. Here, we focused on demonstrating the utility of the microfluidic system for short-read sequencing, however, this technology can be readily implemented to prepare samples for other sequencing instruments including long-read sequencers. Long sequence reads bring advantages in creating *de novo* assemblies and plasmid identification, an appealing aspect for pathogen tracking and characterization. With the capability of our system to serialize mixing and pull-down/purification steps by re-using micro-architectural elements, the platform can also be used for other applications such as RNA sequencing, epigenomic analyses, and perhaps even mass spectroscopy with exactly the same micro-architecture or slightly modified micro-architecture. Ultimately, automated microfluidic platforms may enable the readout of all clinically relevant variation from a single low-quantity sample in one microfluidic system once additional applications are validated (*eg* genome, epigenome, gene expression, proteome, and metabolome).

Supplementary Method 1. Tagmentation (microfluidic 50 pg input method). On-device library construction was carried out according to the following protocol:

1) Pre-treat the surface of the device by flowing Pre buffer (0.5% Kolliphor P188 (Sigma), 0.5% Pluronic F-127 (Sigma), and 5% Tween20 (Sigma)) through the device
   ** Library conversion efficiency improves from ~5% to 15% when the device is pretreated with surfactants[25]
2) Add 4 nL of 10X Tagmentation buffer (one 4 nL segment of the reactor)
3) Add 24 nL of Nextera Enzyme (one 16 nL segment and two 4 nL segments of the reactor)
4) Add 4 nL of wash buffer (10 mM Tris-HCl, 0.5% Tween20) (one 4 nL segment of the reactor)
5) Add 4 nL (one 4 nL segment of the reactor) of 12.5 ng/µL gDNA
6) Mix the reactors with the peristaltic pump for 10 min at 25 C
7) Place the device on a flat top thermo cycler (Bio-Rad) and mix for 12 min at 58 C.
8) Displace 4 nL of the mixture inside the reactors with 2% SDS
9) Mix the reactors for 5 min at 25 C (all mixing performed with three peristaltic pump valves operating at 60 Hz)
10) Incubate at 72 C for 10 min
11) Displace 16 nL of the mixture with binding bead/buffer mixture consisting of 8 x 10^6 M-270 COOH beads (ThermoFisher) and 1.4 x 10^6 6 µm CML beads (ThermoFisher) in 15 µL 2.5 M potassium acetate (Sigma), 36% w/v PEG-8000 (Sigma) in H_2O.
12) Mix the reactors for 15 min at 25 C
13) Strain the mixture through the filter valve unit (top filter valve, retaining the beads in a column)
14) Wash the beads by flushing 200 nL of ethanol through the bead column
15) Air-dry the bead column for 1 min by flowing air through the bead column
16) Elute the DNA by flowing wash buffer through the bead column, storing the eluate in the 20 nL holding tank
17) Discard the beads by opening the filter valves and flushing the filter unit with wash buffer to evacuate beads through the sewer port of each reactor unit
18) Plug 10 µL pipette tips into the output ports and collect the solution in the holding tank by flowing wash buffer (5 µL of solution is collected)
19) The device is cleaned by running cleaning buffer (0.4 M KOH, 10 mM DTT, 0.5% Tween20) through the flow channels for 5 minutes

Supplementary Method 2. Custom optimized lysis and DNA extraction for low-input *E. coli*, *M. tuberculosis*, and iChip micro-colonies (bench-top method).

1) Add 7 µL of cells (~10^4 total cells) to a PCR tube
2) With the tube cap off, the heat for 20 min at 80 C
3) Add 4 µL DNase solution (2 µL dsDNA Fragmentase (New England Biolabs) and 0.5 µL 2 mg/mL BSA (New England Biolabs) in 10 µL P1SK buffer (0.5% vol/vol Tween20, 5% vol/vol Igepal-630 (Sigma), 10 mM $CaCl_2$ (Sigma), 3 mM $MgCl_2$ in Qiagen P1 buffer with RNase))
4) Incubate 30 min at 37 C, then 10 min at 80 C
5) Add 4 µL lysis enzyme mix (1 µL 20% wt/vol SDS, 1 µL 20 mg/mL Proteinase K (Sigma), 2 µL 6 KU/mL Mutanolysin (Sigma), and 2 µL 20 mg/mL Lysozyme (Sigma) in 14.5 µL P1SK buffer)
6) Incubate 25 min at 37 C, 30 min at 56 C, then 10 min at 80 C
7) Mix 12 µL of bead/buffer mixture (8 x 10^6 M-270 COOH beads and 1.4 x 10^6 6 µm CML beads in 15 µL 2.5 M potassium acetate, 36% wt/vol PEG-8000 in H_2O) in each reactor
8) Wash beads two times with 100% ethanol using a magnet

9) Elute with 10 µL of wash buffer
10) Proceed to bench-top tagmentation

Supplementary Method 3. Lysis and DNA extraction for low-input *E. coli*, *M. tuberculosis*, and iChip micro-colonies (microfluidic method)

1) Prepare bead mix on-bench before loading (8×10^6 M-270 COOH beads and 1.4×10^6 6 µm CML beads in wash buffer)
2) Create bead column in the device using the top filter valves
3) Aspirate 7 µL of cells (~10^4 total cells) using a micropipette and plug the loaded pipette tip on the loading port
4) Concentrate (filter) the cells by flushing all 7 µL of the cell mix solution through the on-device bead column
5) Heat shock by incubating 20 minutes at 80 C
6) Add DNase solution to the cells trapped in the on-device bead column
7) Incubate 30 min at 37 C, then 10 min at 80 C
8) Using 16 nL lysis enzyme mix (1 µL 20% SDS, 1 µL 20 mg/mL Proteinase K, 2 µL 6 KU/mL Mutanolysin, and 2 µL 20 mg/mL Lysozyme in 14. 5 µL P1SK buffer), displace the beads and cells in the filter valve section of the device to the reactors
9) Incubate 25 min at 37 C, 30 min at 56 C, then 10 min at 80 C
10) Load 20 nL binding bead/buffer mixture (8×10^6 M-270 COOH beads and 1.4×10^6 6 µm CML beads in 15 µL 2.5 M potassium acetate, 36% wt/vol PEG-8000 in H_2O) in each reactor
11) Mix the reactors for 15 min at 25 C
12) Strain the mixture through the filter valve unit, retaining the beads in a column
13) Wash the beads by flushing 200 nL of ethanol through the bead column
14) Air-dry the bead column for 1 min at 20 psi flow
15) Using 16 nL of wash buffer, load the beads into the reactor
16) Add 4 nL of 10X Tagmentation buffer (one 4 nL segment of the reactor)
17) Add 16 nL of Nextera Tagment DNA Enzyme (one 16 nL segment and two 4 nL segments of the reactor)
18) Proceed to on-device tagmentation

Supplementary Method 4. Base calling and annotating SNPs. The base calling process (Supplementary Figure 10) and commands used to determine genomic diversity between the clinical isolates and soil micro-colony samples were as follows:

1. Read preparation
    a. Trim low quality end of reads: Fastx-Toolkit (v0.0.13)
       > fastx_trimmer -Q33 -f 1 -l 100 -i read.fastq -o out.fastq
    b. Remove Nextera adapter sequence: Cutadapt (v1.8)
       > python cutadapt -a CTGTCTCTTATACACATCT -A CTGTCTCTTATACACA TCT -o read1.fastq -p read2.fastq out1.fastq out2.fastq --minimum-length=5
2. Mapping
    a. Align reads to reference: bwa mem (v0.7.10-r789)
       > bwa mem referencegenome.fasta read1.fastq read2.fastq > aligned_reads.sam
3. SNP identification
    a. Sort aligned bam file: Picard tools (v1.128)
       > java –jar –Xmx16G picard.jar SortSam I= in.sam O= out.bam SO= coordinate
    b. Mark duplicates in the bam file: Picard tools (v1.128)
       > java –jar –Xmx16G picard.jar MarkDuplicates I= in.bam O= out.bam M= out_duplicates

    c. Index reads: Samtools (v1.2)
       > Samtools index in.bam
    d. mpileup to create vcf files (list of potential SNPs) with > Q30 reads: Pilon (v1.8)
       > java –Xmx16G –jar pilon-1.8.jar --genome reference.fasta –fragsin.bam --vcf --output prefix --minqual 30
    e. Omit indels: custom python code (‘step1_indel_remover.py’ and its dependency ‘step1_req_checkDP.awk’) that deletes lines with indel tags in the Pilon generated vcf files
    f. Convert each vcf file to a .mat file compatible for matlab processing using a custom python code (‘step2_vcftomatrix_vcf_v7.py’) that uses regular expression to parse 1) position on the genome, 2) counts for each base, 3) total coverage, 4) mapping quality, 5) allelic fraction, and quality notes (deletion, insertion, and low coverage)
    g. Use custom Matlab code (‘step3_truebase_SNP_finder.m’) to filter and select only reference genome positions that passed our filtering thresholds: coverage > 6, mapping quality > 45, allelic fraction > 0.82, and is not an indel site (determined by ‘step4_concVSdisc_snp_by_AF.m’ and ‘step5_ROCbyAF.m’)
    h. Use custom Matlab code (‘step6_intra_SNP_finder.m’) to compare consensus basecalls from each isolate to each other for all reference genome positions and find where the quality filtered consensus basecalls of each sample are different

4. Annotation (clinical *Pseudomonas* isolates)
    a. Use vcfannotator (http://sourceforge.net/projects/vcfannotator) with the PA14 GFF3 annotation file to annotate the SNPs
       > VCF_to_annotated_SNP_report.pl --gff3 annotationg.gff3 --genome genome.fasta --vcf in.vcf -X out.txt > out.vcf

Supplementary Method 5. SNP calling / error filtering threshold selection (*Pseudomonas*). In determining the genomic diversity among the clinical isolates, the imperfect alignment and accuracy of sequence reads required us to balance the need to make base calls for most positions (sensitivity) with making a high fraction of correct calls (specificity). To do so, we take into account statistics on the read and mapping quality, coverage at each genomic position, and co-occurrence between triplicate samples.

First, we list potential SNPs in variant call format by aligning de-duplicated and quality-trimmed-sequencing reads to the PA14[26] GenBank reference genome [NC_008463.1]. Then, we compare whether SNPs were shared among triplicates. Since each triplicate replicate set originated from the same gDNA source, discordance almost certainly indicates the presence of an error that occurred during library construction or sequencing.

We define SNPs as positions that differ from the reference genome. “Concordant SNPs” are SNP calls that agree among all three replicates, and “discordant SNPs” disagree in at least one of the three replicates (Figure 2b; table below). Note that we avoid making indel calls. This class of calls is often more ambiguous than substitution calls, particularly when there are significant differences between the reference sequence and the strains under analysis.

To quality filter our data, we set thresholds on coverage (6) and mapping quality (45). This coverage threshold is commonly used in other SNP analysis studies[15]. The mapping quality threshold was chosen based on the distribution of mapping qualities (Supplementary Figure 14) of the *P. aeruginosa* sequence data and the distribution of mapping qualities observed for positive-control antibiotic resistance SNPs, whose loci all showed mapping quality > 55.

Our objective was to minimize errors that arose during sample preparation by selecting a filtering threshold that minimizes the discordant SNPs across triplicate libraries without losing sensitivity. To do so, we varied a third threshold on the quality-adjusted[27] allelic fraction (AF = variant base counts / total base counts). We set the filtering threshold at AF = 0.82 at which the fraction of discordant SNPs was set to zero, while the fraction of concordant SNPs was maximized at 0.998 (Figure 2c) across triplicate sample sets from each of our six subjects.

Replicate 1	T	A	A	A	NC	A	T	A	NC
Replicate 2	T	A	A	T	T	A	NC	NC	NC
Replicate 3	T	A	T	T	T	NC	NC	NC	NC
Reference genome	T	T	T	T	T	T	T	T	T
**Call made**	**-**	**Conc**	**Disc**	**Disc**	**-**	**-**	**-**	**-**	**-**

* “NC” are “no calls” or bases that fell below quality thresholds. “Conc”, and “Disc” are concordant, and discordant sites respectively. “-“ are genome positions that are removed from the analysis. Note that only reference-variant bases are included in the analysis.

Supplementary Method 6. Specificity and sensitivity of SNP calling. To measure the sensitivity and specificity of our base calling procedure at 20X and 50X coverage, we sequenced one sample (P01-04) to a much higher depth (340X) for comparison. We reasoned with much higher coverage we could identify SNPs that were lost to low coverage and erroneous SNP calls due to errors in library construction or sequencing.

We prepared a conventional bench-top library from a clinical *P. aeruginosa* isolate sample (P01-04) and sequenced it to 340X depth. To measure SNP base calling accuracy in both bench-top and device libraries, we prepared two more bench-top libraries, and three device libraries from the same sample. All libraries were sequenced to 20X depth in a single MiSeq run (2x150).

We sequenced three replicates of each sample to compare the base calling accuracy in reads pooled from replicate libraries to a single 50X library. To do so, we merged the three 20X device libraries into a single fastq file to enable an equivalent 50X library to be produced. For comparison, we also randomly sampled reads from the single 340X bench-top library to produce a 50X library.

We defined true positives (TP) as correctly identified SNPs compared to the ground truth, true negatives (TN) as correctly identified non-variant bases, and false positives (FP) as mistakenly called SNPs. False negatives (FN) encompass both incorrectly identified bases and true SNPs that were uncalled due to low coverage (table below).

All data was restricted to positions where calls in both the test library and ground truth library passed the quality thresholds established earlier.

Test library	T	A	A	T	T	A	NC	NC	NC
Ground truth (340X library)	T	T	A	A	NC	NC	NC	T	A
Reference genome	T	T	T	T	T	T	T	T	T
Call made	TN	FP	TP	FN	-	-	-	-	FN

* “NC” are “no calls” or bases that fell below quality thresholds. “-“ are genome positions removed from the analysis.

To determine if we achieved optimal specificity and sensitivity for base calling given the thresholds from our concordance analysis (Figure 2c), we examined performance as we varied the AF threshold to produce ROC curves. The other quality thresholds are held constant (coverage at 6 and mapping quality at 45).

Supplementary Method 7. *De novo* assembly. We used SPAdes[2] genome assembler (v3.6.2) to *de novo* assemble sequencing reads. The assembled contigs were filtered for >800 bp length and >2 coverage.

```
> spades.py -k 55, 71, 85 --pe1-1 read1.fastq --pe1-2 read2.fastq --careful -o spades_output
```

Supplementary Method 8. Soil micro-colony phylotyping. We used the following Phylosift[3] (v1.0.1) commands to phylotype the soil micro-colonies we cultured in the iChip.

```
> phylosift all --threads 7 contig.fasta --output outputdir
> phylosiftdir/bin/guppy tog --out-dir outdir input.jplace
```

Supplementary Method 9. Human DNA contamination level determination. We determined the human DNA contamination level using DeconSeq (v0.4.3; https://sourceforge.net/projects/deconseq/files/).

```
> perl deconseq.pl -f reads.fastq -dbs hsref -out_dir outdir
```

Supplementary Method 10. Duplication rate determination for library complexity estimation. Duplicate reads for *E. coli*, clinical *P. aeruginosa*, and *M. tuberculosis* were identified by searching for reads with identical mapped starting and ending positions (Picard-tools).

```
> java –jar –Xmx16G picard.jar MarkDuplicates I= in.bam O= out.bam M= out_duplicates
```

Since no reference genomes were used in our soil micro-colony analysis, we determined the duplication rate by comparing sequences of each read to another. We used PRINSEQ[28] (v0.20.4) for these samples:

```
> perl prinseq-lite.pl -fastq read1.fastq -fastq2 read2.fastq -phred64 -derep 1 -log out.txt
```

The duplication rate was used in turn to estimate the library complexity (Supplementary Note 3).

Supplementary Method 11. Secondary metabolite profiling of the soil micro-colonies. The secondary metabolite gene-cluster prediction was performed on our *de novo* assembled contigs using Anti-smash 3.0[29] with default settings.

Supplementary Method 12. Pangenome analysis. The clinical *P. aeruginosa* isolate samples were profiled functionally using HUMAnN2 (http://huttenhower.sph.harvard.edu/humann2/manual)[30]. Briefly, HUMAn2 maps sequence reads to translated protein-coding sequences of annotated genes[1214]. All hits are weighted based on alignment quality and sequence length, with per-species and unclassified hits combined to produce community totals for each protein family (in addition to species-stratified totals) in RPK (reads per kilobase) units. RPK units were further normalized to RPKM units (reads per kilobase per million sample reads) to account for variation in read depth across samples. Here, we focused only on reads mapping onto *P. aeruginosa* pangenome.

Functional profiling of the *P. aeruginosa* clinical isolate samples yielded abundance measurements for 10,510 microbial gene families. We trained a Random Forest classifier to

identify gene families that were most informative for separating the subjects. We identified 1,021 gene families that had a variable importance score greater than 1.0 (scaled mean decrease in accuracy was used as the variable importance score). To make downstream analysis of these families more informative, we grouped gene family abundance of these 1,021 gene families into broader functional categories based on annotations between UniProt proteins[31] (of which our ~11 million UniRef50 protein families are a subset) and gene ontology (GO)[32,13]. We allowed protein annotations to propagate upward through the child-parent relationships among GO terms. For example, a protein annotated with the term “carbohydrate metabolism” was automatically annotated to that term’s less specific parent, “primary metabolic process”. Following previous work[33,34], we isolated a subset of “informative” GO terms, defined as terms associated with >k proteins for which no descendant term was associated with >k proteins (here, k = 50,000, which equates to ~1 out of every 50,000 UniRef50 protein families). This procedure yielded a comprehensive but manageable set of 34 GO terms for subsequent analysis. By the nature of their construction, informative GO terms tend to provide more resolution for well-conserved and well-studied processes (which are annotated to many proteins) and place less focus on highly specific processes associated with only a small number of proteins.

Supplementary Method 13. Gene set enrichment analysis. In order to study functional differences in different *P. aeruginosa* isolates, we divided gene families from HUMAnN2 pipeline (see above) into core and variable genome. Gene families that were present in all replicates were assigned into the core genome and all other gene families into the variable genome. With another set of “informative” GO terms, now k = 1,000 (see above), we mapped gene families to 350 molecular function and 485 biological process GO categories. We then tested these GO categories for over-presentation in variable genome using a test of proportions (prop.test function in R). Nominal p-values were corrected using the Benjamini-Hochberg false discovery rate method[35].

Supplementary Method 14. *Pseudomonas aeruginosa* isolate variable genome novel content analysis. The content of the ~10% region of the genome of our *P. aeruginosa* isolate data that was not represented in the PA14 reference were analyzed by performing metagenomic analysis with Metaphlan 2.0[24] (http://huttenhower.sph.harvard.edu/metaphlan) on the reads that did not align to the PA14 reference genome. Metaphlan uses the Bowtie2[36] aligner to quantify reads from different organisms in each sample by mapping the reads to the Metaphlan marker database (which includes phage sequences). The process is as follows:

1) Start with PA14 reference genome and aligned/de-duplicated BAM files
2) Use Samtools to select only the unmapped reads
```
> samtools view –bhf 4 in.bam > out.bam
> java –jar –Xmx16G picard.jar SamToFastq I= in.bam F= out1.fastq F2= out2.fastq
> cat out1.fastq out2.fastq > out.fastq
```
3) Run Metaphlan 2.0 to quantify reads mapping to taxonomic marker sequences
```
> python metaphlan2.py in.fastq –mpa_pkl mpa_v20_m200.pkl –bowtie2db mpa_v20_m200 –bowtie2out out.bowtie2.bz2 –stat_q 0.05 –nproc 7 -input_type fastq > out.txt
> python merge_metaphlan_tables.py *.txt > merged_out.txt
```
4) Cluster marker hit profiles by samples (Supplementary Figure 19)
```
> python metaphlan_hclust_heatmap.py -c bbcry --top 50 --font_size 8 --minv 0.01 -s log -f euclidean -d euclidean –in in.txt --out out.png -c jet
```

List of supplementary files (publicly available on https://sourceforge.net/projects/sk-dev-cad-analysis-software/files/Kim_supplementaryfiles.zip /download)

1) Microdevice design file (autocad)
   1ShrinkSKv8.15C-4096.Unionstretch.dwg
2) Custom scripts to ready the vcf for parsing
   step1_indel_remover.py; step1_req_checkDP.awk
3) Custom script to parses vcf to .mat (python)
   step2_vcftomatrix_vcf_v7.py
4) Custom script to filter mapped bases and SNPs compared to the reference genome (matlab)
   step3_truebase_SNP_finder.m
5) Custom script to generate concordance vs discordance plot (matlab)
   step4_concVSdisc_snp_by_AF.m
6) Custom script to generate receiver operator characteristic plot (matlab)
   step5_ROCbyAF.m
7) Custom script to find SNPs between samples (matlab)
   step6_intra_SNP_finder.m

Supplementary References

1. Melin, J. & Quake, S. R. Microfluidic large-scale integration: the evolution of design rules for biological automation. *Annu. Rev. Biophys. Biomol. Struct.* **36,** 213–31 (2007).

2.

Bankevich, A. *et al.* SPAdes: a new genome assembly algorithm and its applications to single-cell sequencing. *J. Comput. Biol.* **19,** 455–77 (2012).

3. Darling, A. E. *et al.* PhyloSift: phylogenetic analysis of genomes and metagenomes. *PeerJ* **2,** e243 (2014).

4. DeAngelis, M. M., Wang, D. G. & Hawkins, T. L. Solid-phase reversible immobilization for the isolation of PCR products. *Nucleic Acids Res.* **23,** 4742–3 (1995).

5. Li, H. Mathematical Notes on SAMtools Algorithms. (2010). at <https://www.broadinstitute.org/gatk/media/docs/Samtools.pdf>

6. White, R. A., Blainey, P. C., Fan, H. C. & Quake, S. R. Digital PCR provides sensitive and absolute calibration for high throughput sequencing. *BMC Genomics* **10,** 116 (2009).

7. Bentley, D. R. *et al.* Accurate whole human genome sequencing using reversible terminator chemistry. *Nature* **456,** 53–9 (2008).

8. Robasky, K., Lewis, N. E. & Church, G. M. The role of replicates for error mitigation in next-generation sequencing. *Nat. Rev. Genet.* **15,** 56–62 (2014).

9. Costello, M. *et al.* Discovery and characterization of artifactual mutations in deep coverage targeted capture sequencing data due to oxidative DNA damage during sample preparation. *Nucleic Acids Res.* **41,** e67 (2013).

10. Ozer, E. A., Allen, J. P. & Hauser, A. R. Characterization of the core and accessory genomes of Pseudomonas aeruginosa using bioinformatic tools Spine and AGEnt. *BMC Genomics* **15,** 737 (2014).

11. Bauer, A. W., Kirby, W. M., Sherris, J. C. & Turck, M. Antibiotic susceptibility testing by a standardized single disk method. *Am. J. Clin. Pathol.* **45,** 493–6 (1966).

12. Huang, K. *et al.* MetaRef: a pan-genomic database for comparative and community microbial genomics. *Nucleic Acids Res.* **42,** D617-24 (2014).

13. The Gene Ontology Consortium. Gene Ontology Consortium: going forward. *Nucleic Acids Res.* **43,** D1049-1056 (2014).

14. Suzek, B. E., Wang, Y., Huang, H., McGarvey, P. B. & Wu, C. H. UniRef clusters: a comprehensive and scalable alternative for improving sequence similarity searches. *Bioinformatics* **31,** 926–32 (2015).

15. Lieberman, T. D. *et al.* Genetic variation of a bacterial pathogen within individuals with cystic fibrosis provides a record of selective pressures. *Nat. Genet.* **46,** 82–7 (2014).

16. Romling, U., Wingender, J., Muller, H. & Tummler, B. A major Pseudomonas aeruginosa clone common to patients and aquatic habitats. *Appl. Envir. Microbiol.* **60,** 1734–1738 (1994).

17. Darch, S. E. *et al.* Recombination is a key driver of genomic and phenotypic diversity in a Pseudomonas aeruginosa population during cystic fibrosis infection. *Sci. Rep.* **5,** 7649 (2015).

18. Smith, E. E. *et al.* Genetic adaptation by Pseudomonas aeruginosa to the airways of cystic fibrosis patients. *Proc. Natl. Acad. Sci. U. S. A.* **103,** 8487–92 (2006).

19. Struelens, M. J., Schwam, V., Deplano, A. & Baran, D. Genome macrorestriction analysis of diversity and variability of Pseudomonas aeruginosa strains infecting cystic fibrosis patients. *J. Clin. Microbiol.* **31,** 2320–2326 (1993).

20. Workentine, M. L. *et al.* Phenotypic heterogeneity of Pseudomonas aeruginosa populations in a cystic fibrosis patient. *PLoS One* **8,** e60225 (2013).

21. Folkesson, A. *et al.* Adaptation of Pseudomonas aeruginosa to the cystic fibrosis airway: an evolutionary perspective. *Nat. Rev. Microbiol.* **10,** 841–51 (2012).

22. Lieberman, T. D. *et al.* Parallel bacterial evolution within multiple patients identifies candidate pathogenicity genes. *Nat. Genet.* **43,** 1275–80 (2011).

23. Kung, V. L., Ozer, E. A. & Hauser, A. R. The accessory genome of Pseudomonas aeruginosa. *Microbiol. Mol. Biol. Rev.* **74,** 621–41 (2010).

24. Segata, N. *et al.* Metagenomic microbial community profiling using unique clade-specific marker genes. *Nat. Methods* **9,** 811–4 (2012).

25. Luk, V. N., Mo, G. C. & Wheeler, A. R. Pluronic additives: a solution to sticky problems in digital microfluidics. *Langmuir* **24,** 6382–9 (2008).

26. Winsor, G. L. *et al.* Pseudomonas Genome Database: improved comparative analysis and population genomics capability for Pseudomonas genomes. *Nucleic Acids Res.* **39,** D596-600 (2011).

27. Walker, B. J. *et al.* Pilon: an integrated tool for comprehensive microbial variant detection and genome assembly improvement. *PLoS One* **9,** e112963 (2014).

28. Schmieder, R. & Edwards, R. Quality control and preprocessing of metagenomic datasets. *Bioinformatics* **27,** 863–4 (2011).

29. Weber, T. *et al.* antiSMASH 3.0--a comprehensive resource for the genome mining of biosynthetic gene clusters. *Nucleic Acids Res.* **43,** W237-243 (2015).

30. Abubucker, S. *et al.* Metabolic reconstruction for metagenomic data and its application to the human microbiome. *PLoS Comput. Biol.* **8,** e1002358 (2012).

31. The UniProt Consortium. UniProt: a hub for protein information. *Nucleic Acids Res.* **43,** D204-212 (2014).

32. Dimmer, E. C. *et al.* The UniProt-GO Annotation database in 2011. *Nucleic Acids Res.* **40,** D565-70 (2012).

33. Zhou, X., Kao, M.-C. J. & Wong, W. H. Transitive functional annotation by shortest-path analysis of gene expression data. *Proc. Natl. Acad. Sci. U. S. A.* **99,** 12783–8 (2002).

34. Huang, Y. *et al.* Systematic discovery of functional modules and context-specific functional annotation of human genome. *Bioinformatics* **23,** i222-9 (2007).

35. Benjamini, Y. & Hochberg, Y. Controlling The False Discovery Rate - A Practical And Powerful Approach To Multiple Testing. *J. R. Stat. Soc. Ser. B Methodol.* **57,** 289–300 (1995).

36. Langmead, B. & Salzberg, S. L. Fast gapped-read alignment with Bowtie 2. *Nat. Methods* **9,** 357–9 (2012).
